# Spontaneous Mutations in HIV-1 Gag, Protease, RT p66 in the First Replication Cycle and How They Appear: Insights from an In Vitro Assay on Mutation Rates and Types

**DOI:** 10.3390/ijms22010370

**Published:** 2020-12-31

**Authors:** Joshua Yi Yeo, Darius Wen-Shuo Koh, Ping Yap, Ghin-Ray Goh, Samuel Ken-En Gan

**Affiliations:** 1 Bioinformatics Institute, A*STAR, 30 Biopolis Street, #07-01 Matrix, Singapore 138671, Singapore; yeoyj@bii.a-star.edu.sg (J.Y.Y.); kohwsd@bii.a-star.edu.sg (D.W.-S.K.); yapping72@gmail.com (P.Y.); ghinray04@gmail.com (G.-R.G.); 2 Experimental Drug Development Centre, A*STAR, 10 Biopolis Road Chromos #05-01, Singapore 138670, Singapore; 3 p53 Laboratory, A*STAR, 8A Biomedical Grove, #06-04/05 Neuros/Immunos, Singapore 138648, Singapore

**Keywords:** HIV, Gag, protease, reverse transcriptase, mutation rate, drug resistance

## Abstract

While drug resistant mutations in HIV-1 are largely credited to its error prone HIV-1 RT, the time point in the infection cycle that these mutations can arise and if they appear spontaneously without selection pressures both remained enigmatic. Many HIV-1 RT mutational in vitro studies utilized reporter genes (LacZ) as a template to investigate these questions, thereby not accounting for the possible contribution of viral codon usage. To address this gap, we investigated HIV-1 RT mutation rates and biases on its own Gag, protease, and RT p66 genes in an in vitro selection pressure free system. We found rare clinical mutations with a general avoidance of crucial functional sites in the background mutations rates for Gag, protease, and RT p66 at 4.71 × 10^−5^, 6.03 × 10^−5^, and 7.09 × 10^−5^ mutations/bp, respectively. Gag and p66 genes showed a large number of ‘A to G’ mutations. Comparisons with silently mutated p66 sequences showed an increase in mutation rates (1.88 × 10^−4^ mutations/bp) and that ‘A to G’ mutations occurred in regions reminiscent of ADAR neighbor sequence preferences. Mutational free energies of the ‘A to G’ mutations revealed an avoidance of destabilizing effects, with the natural p66 gene codon usage providing barriers to disruptive amino acid changes. Our study demonstrates the importance of studying mutation emergence in HIV genes in a RT-PCR in vitro selection pressure free system to understand how fast drug resistance can emerge, providing transferable applications to how new viral diseases and drug resistances can emerge.

## 1. Introduction

RNA viruses have a higher likelihood of genetic changes, leading to species jump [1] and efficient spread among humans [2]. Among RNA viruses, the human immunodeficiency viruses HIV-1 and HIV-2 are reported to be zoonotic transmissions of the simian immunodeficiency viruses (SIV) [3,4,5].

Within HIV-1, the Gag and protease proteins play crucial roles in viral assembly and maturation of infectious virions [6]. Protease cleaves Gag and Pol polyproteins into functional subunits [7] and this is prevented by protease inhibitors (PI) which compete with Gag for the active site [8,9]. In emerging PI resistance, mutations on viral protease reduce affinity to PIs [9,10], and the gradual accumulation of many such resistance mutations [11,12,13] induced by HIV-1 RT [14,15], eventually limit clinical drug selection due to cross-resistances [9]. At the same time, mutations on the substrate Gag are reported to compensate for reduced viral fitness [9,10,15,16,17], working synergistically with protease mutations to overcome PIs [17,18,19,20,21]. 

In the causative spotlight in HIV drug resistance mutations is the error prone enzyme reverse transcriptase (RT), an asymmetric heterodimer of the p66 and p51 subunits [22]. The p66 subunit catalyzes DNA polymerisation and cleaves the RNA of the RNA/DNA duplex [23,24] while the p51 subunit plays a supportive role to p66 [25]. 

**Figure 1:**
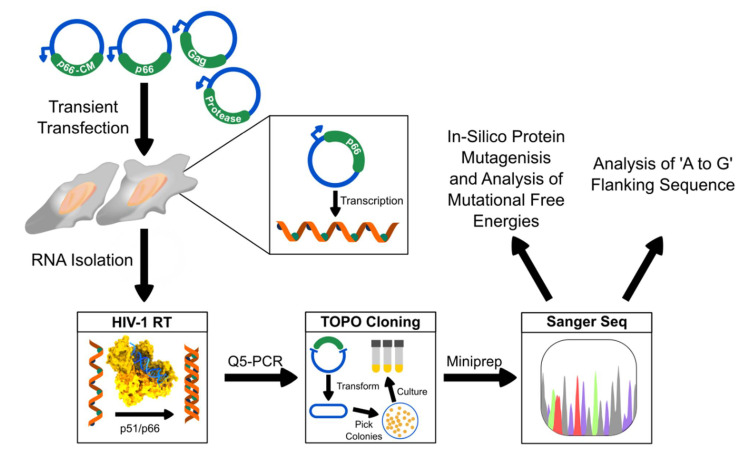
A schematic diagram outlining the workflow of the in vitro selection pressure free HIV-RT fidelity assay. Vectors containing HIV-1 Gag, protease, RT p66, and Codon Mutated RT were transiently transfected into EXPI293F cells. Total RNA was extracted and subjected to HIV-RT cDNA synthesis followed by Q5 PCR amplification for TOPO blunt-end cloning and the sequence that was analyzed. Mutants were subjected to computational analysis with respect to their mutational free energies and flanking sequences of ‘A to G’.

In the investigation of HIV mutations, in-depth analysis of RT mutations is required. However, most previous studies utilized reporter genes such as LacZ and not HIV genes for the analysis of mutations. This leaves a gap in understanding the contribution of HIV gene-specific sequences and codon usage in mutational hotspots, as well as type of mutations and when they can emerge in the infection cycle [26]. To fill this gap, we developed a low biosafety level in vitro based assay (see Figure 1 for a schematic representation) without translational, immune, and drug selection pressures to characterize the innate basal HIV-1 RT mutations and biases on HIV-1 Gag, protease, and RT p66 genes. As a control and further investigation to the natural codon usage, we also created a silent codon mutated variant of RT p66. Together, our findings shed light on the emergence of drug resistance mutations, the native rate, where they appear, and the associated biases and trends that would be useful for the design of future drug interventions. 

## 2. Results

### 2.1. Characterization of HIV-1 Gag, Protease, RT p66, and Codon Mutated RT p66 Mutant Variants 

**Figure 2:**
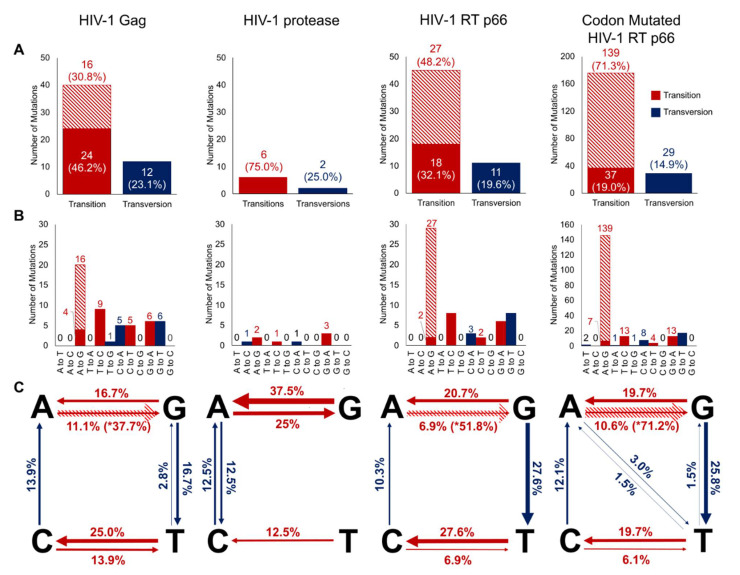
Generated HIV-1 Gag, protease, RT p66, and Codon Mutated RT p66 nucleotide substitution mutations using HIV-1 RT. (**A**) Bar chart of transitions and transversions mutations observed in HIV-1 Gag (*n* = 52), protease (*n* = 8), RT p66 (*n* = 56) and codon mutated p66 (*n* = 205). Transitions and transversions are shown in red and blue, respectively. ‘A to G’ hypermutations are shown separately as diagonal stripes. (**B**) Bar chart of substitution mutations observed in HIV-1 Gag, protease, RT p66, and codon mutated p66 sequences. (**C**) Schematic diagram of relative nucleotide substitution frequencies on the respective HIV-1 genes (expressed as a percentage).

**Figure 3:**
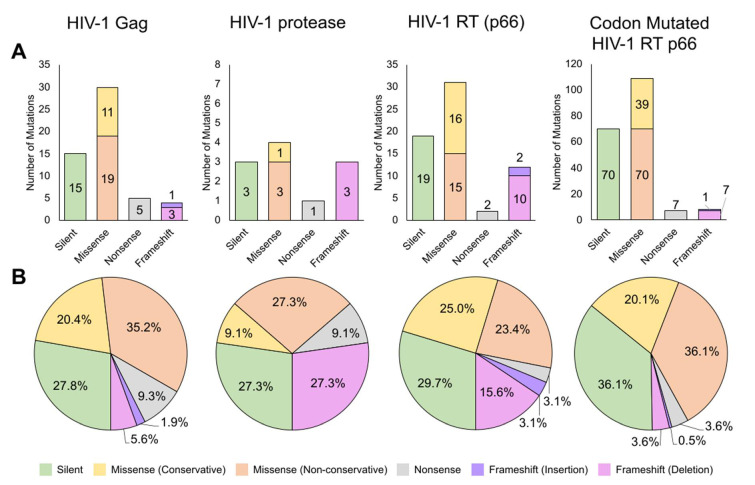
Generated amino acid mutations using HIV-1 RT. (**A**) Bar chart of amino acid mutations observed in HIV-1 Gag (*n* = 54), protease (*n* = 11), RT p66 (*n* = 64), and codon mutated RT p66 (*n* = 194). (**B**) Pie chart of the relative amino acid mutation frequencies (expressed as a percentage). For the codon mutated RT p66, two variants (20 and 80 a, b) containing deletions without frameshifts are not shown. Silent, missense (conservative), missense (non-conservative), nonsense, frameshift (insertion), and frameshift (deletion) mutations are shown in green, yellow, orange, gray, purple, and pink, respectively.

‘A to G’ mutations were classified as hypermutations when multiple such mutations were found on the same gene clone, especially given that mutations should statistically be evenly distributed. 801 HIV-1 Gag sequences were generated and calculated to show a mutation rate of 3.36 × 10^−5^ mutations/bp and at 4.71 × 10^−5^ mutations/bp when including ‘A to G’ hypermutations (see Table 1). More transitions (*n* = 24 excluding ‘A to G’ hypermutations and *n* = 40 including) than transversions (*n* = 12) were found (Figure 2A). ‘A to G’ substitutions were found to be the most frequent (*n* = 20, 37.7%) when including ‘A to G’ hypermutations, but when excluded, ‘T to C’ substitutions were the most frequent (*n* = 9, 25.0%), followed by ‘G to A’ and ‘G to T’ (*n* = 6, 16.7%), ‘C to A’ and ‘C to T’ (*n* = 5, 13.9%), ‘A to G’ (*n* = 4, 11.1%), and ‘T to G’ (*n* = 1, 2.8%), with several substitutions types not observed (see Figure 2B,C). Amino acid analysis showed missense mutations (*n* = 30, 55.6%) to occur at approximately twice the frequency of silent mutations (*n* = 15, 27.8%, see Figure 3) while nonsense mutations (*n* = 5, 9.3%) and frameshift mutations (*n* = 4, 7.5%) had lower occurrences (see Supplementary Table S1 for full list of mutations). 

For HIV-1 protease, 640 sequences were generated with a calculated mutation rate of 6.03 × 10^−5^ mutations/bp (see Table 1). No ‘A to G’ hypermutations were found for protease. As with Gag, there were more transition mutations (*n* = 6) than transversions (*n* = 2) as shown in Figure 2A. ‘G to A’ substitutions were found to be the most frequent (*n* = 3, 37.5%), followed by ‘A to G’ (*n* = 2, 25%), ‘A to C’, ‘T to C’, and ‘C to A’ (*n* = 1, 12.5%), with several substitutions not observed (see Figure 2B,C). Amino acid analysis showed that missense mutations (*n* = 4, 36.4%) occurred at approximately 1.3 times the frequency of silent mutations (*n* = 3, 27.3%) and frameshift mutations (*n* = 3, 27.3%), whereas nonsense mutations (*n* = 1, 9.1%) were of lower occurrences (see Figure 3, Supplementary Table S2 for full list). 

From 571 HIV-1 RT p66 subunit sequences, we calculated a mutation rate of 4.27 × 10^−5^ mutations/bp when excluding ‘A to G’ hypermutations and 7.09 × 10^−5^ mutations/bp when including them (see Table 1). As with Gag and protease, there were more transition mutations (*n* = 18 excluding ‘A to G’ hypermutations, 45 including ‘A to G’ hypermutations) than transversions (*n* = 11), as shown in Figure 2A. ‘A to G’ substitutions were found to be the most frequent (*n* = 29, 51.8%) only when including ‘A to G’ hypermutations. When excluded, ‘T to C’ and ‘G to T’ substitutions were instead the most frequent (*n* = 8, 27.6%), followed by ‘G to A’ (*n* = 6, 20.7%), ‘C to A’ (*n* = 4, 12.5%), and ‘A to G’ and ‘C to T’ (*n* = 2, 6.9%). There were several substitution types not observed (see Figure 2B,C). Analysis after translation found similar trends for both Gag and protease where missense mutations (*n* = 31, 48.4%) occurred at 1.6 times the frequency of silent mutations (*n* = 19, 29.7%), as shown in Figure 3. Nonsense mutations (*n* = 2, 3.1%) and frameshift mutations (*n* = 12, 18.7%) were of lower occurrences (Supplementary Table S3). 

A total of 700 codon mutated p66 sequences gave a calculated mutation rate of 6.53 × 10^−5^ mutations/bp when excluding ‘A to G’ hypermutations and 1.88 × 10^−4^ mutations/bp when including them (see Table 1). In the same trend with Gag, protease, and wild-type p66, there were more transition mutations (*n* = 37 excluding ‘A to G’ hypermutations, 176 including ‘A to G’ hypermutations) than transversions (*n* = 29) in codon mutated p66 (Fiure 2A). Of the same trend with wild-type p66, ‘A to G’ substitutions were found to be the most frequent (*n* = 146, 71.2%) only when including ‘A to G’ hypermutations, and when excluded, ‘G to T’ substitutions were found to the most frequent (*n* = 17, 25.8%), followed by ‘G to A’ (*n* = 13, 19.7%) and ‘T to C’ (*n* = 13, 19.7%), ‘A to G’ (*n* = 7, 10.6%), ‘C to A’ (*n* = 8, 12.1%), ‘C to T’ (*n* = 4, 6.1%), ‘A to T’ (*n* = 2, 3.0%), and ‘T to A’ and ‘T to G’ substitutions (*n* = 1, 1.5%), with the absence of the following substitutions: ‘A to C’, ‘C to G’ and ‘G to C’ (see Figure 2B,C). Amino acid analysis showed agreement in trends with the other HIV-1 genes where missense mutations (*n* = 109, 56.2%) occurred approximately 1.6 times the frequency of silent mutations (*n* = 70, 36.1%, see Figure 3). Nonsense mutations (*n* = 7, 3.6%) and frameshift mutations (*n* = 8, 4.1%) were at lower occurrences (Supplementary Table S4). 

### 2.2. Mutation Rates of the Domains of HIV-1 Gag, Protease and RT p66

**Figure 4:**
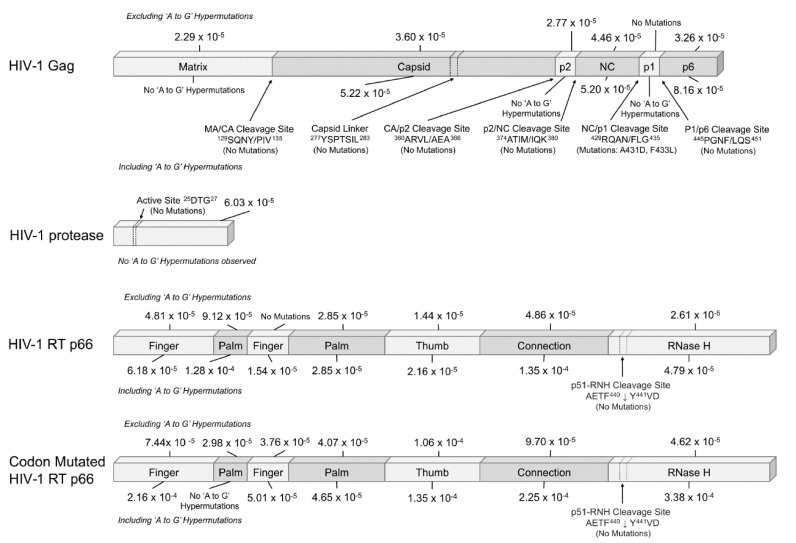
Mutation rates (mutations/bp) calculated in the domains of HIV-1 Gag, protease, RT p66, and Codon Mutated RT p66. Mutation rates were calculated as the ratio of number of mutations observed and the total number of nucleotide bases. Mutation rates were counted separately when including and excluding ‘A to G’ hypermutations. Truncations that span across multiple domains were excluded in calculations. The mutation rates of the respective regions are also shown.

The HIV-1 genes were further analyzed by their respective domains and the mutation rates calculated separately (see Figure 4). Within Gag, the overall mutation rates throughout the domains were within a narrow range of 2.29 to 4.46 × 10^−5^ mutations/bp when excluding ‘A to G’ hypermutations but increased to 8.16 × 10^−5^ mutations/bp when including them. ‘A to G’ hypermutations were found in the capsid (CA), nucleocapsid (NC), and p6 subunits, but not in the matrix (MA), p2 and p1 domains. Mutations were not observed in the MA/CA ^129^SQNY/PIV^135^, CA/p2 ^360^ARVL/AEA^366^, p2/NC ^374^ATIM/IQK^380^, P1/p6 ^445^PGNF/LQS^451^ cleavage sites and the ^277^YSPTSIL^283^ capsid linker. However, a couple of mutations (A431D and F433L) occurred in the NC/p1 ^429^RQAN/FLG^435^ cleavage site.

For HIV-1 protease, there were no ‘A to G’ hypermutations and no mutations in the active site ^25^DTG^27^. For RT p66, when excluding hypermutations, there was a range of 0 to 9.12 × 10^−5^ mutations/bp, which increased to 1.35 × 10^−4^ mutations/bp when including them. Despite the 2nd finger domain being of similar length as the 1st palm segment, only ‘A to G’ hypermutations were observed in the 2nd finger without other mutations. No mutations were observed in the RT p51 and RNase H domain (p51-RNH) cleavage site AETF^440^↓Y^441^VD or in the catalytic triad: D110, D185, and D186. For the codon mutated p66, the mutation rate was in the range of 2.98 × 10^−5^–1.06 × 10^−4^ mutations/bp when excluding hypermutations and 3.38 × 10^−4^ mutations/bp when including them. Contrary to the wild-type p66, there were mutations observed in the 2nd finger domain, suggesting that codon usage of the wild-type 2nd finger domain resisted mutations with the exception of ‘A-G’ hypermutations. Similarly, no mutations were observed in the p51-RNH cleavage site AETF^440^↓Y^441^VD despite the different codon usage. Interestingly, ‘A to G’ hypermutations were not observed in the 1st palm region of the codon mutated p66. 

### 2.3. Flanking Sequences of ‘A to G’ Mutation Sites 

**Figure 5:**
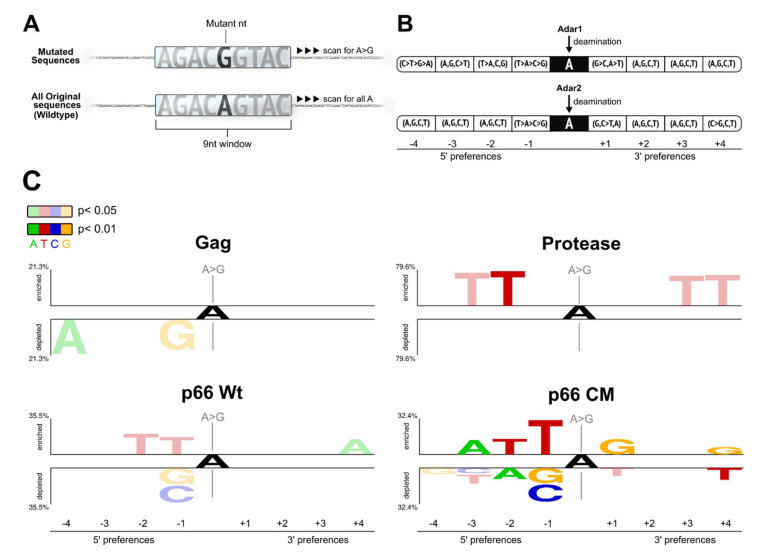
Two Sample logo analysis of HIV-1 Gag, protease, RT p66 (p66 Wt) and Codon Mutated RT (p66 CM). (**A**) The flanking sequence preferences 4 nt upstream and downstream of all ‘A to G’ mutations were determined from the identified adenosine mutations within a 9 nt sliding window. (**B**) A re-representation of the result from [28] on the studied neighbor sequence preferences of ADAR1 and 2 isoforms. Base preferences are shown with inequality signs, where the identity of the base did not significantly contribute to the editing of the 4 bases are delimited by commas. (**C**) Two Sample Logos analysis of the flanking sequences around the ‘A to G ‘mutation sites. Only significantly enriched and depleted neighboring nucleotides flanking A to G mutations are shown. Bases were colored opaque when *p* < 0.01, and translucent when *p* < 0.05. Statistics: two sample *t*-test without Bonferroni correction.

To explore the possible influence of host defense deaminases e.g., ADAR in causing ‘A to G’ hypermutations, we analyzed the flanking 4 nucleotides (nt) of all the detected ‘A to G’ mutations using the two-sample logo analysis (Figure 5A,C) for comparisons with those in the study by Eggington and colleagues to map the ADAR neighboring sequence preferences [28].

Analysis of the Gag sequences showed significant depletions of ‘A’ at −4 and ‘G’ at −1 nt positions upstream of the ‘A to G’ mutation sites (with *p* < 0.05), which are in agreement with the known ADAR neighbor sequence preferences [28], see Figure 5B). Using the Two Sample Logo of multiple and single substitution mutations, there was an enrichment of ‘C’ at positions +1, +4 (both with *p* < 0.05), and −4 nt (*p* < 0.01) (Supplementary Figure S1), with the known two (+1 and +4 nt) positions concurring with ADAR neighbor sequence preferences [28], Figure 5B). For protease, only the ‘T’ enrichment at the −2 nt (Figure 5B) is in agreement with previously reported ADAR neighbor sequence preferences.

Sequence analysis of the wild-type p66 ‘A to G’ mutations reflected the signatures of ADAR sites found by Eggington and colleagues [28], with enrichments of ‘T’ over ‘G’ and ‘C’ at the positions −1, and also at −2 positions, whereas the enrichment of ‘A’ occurred at the +4 positions (*p* < 0.05), see Figure 5C (bottom left panel). For the codon mutated p66, there were enrichments of ‘T’ at positions −1, −2, of ‘A’ at −3 position, and of ‘G’ at the +1 and +4 nt positions (all with *p* < 0.01). 

It is also noticed that for both the p66 sequences, the large representation of multiple mutation variants masked single substitution variants with ADAR neighbor sequence preferences found in the enrichments of ‘T’ at the −1 and −2 nt positions of the 3′ end (Supplementary Figure S1). 

### 2.4. Effects of ‘A to G’ Mutations on Protein Thermostability 

**Figure 6:**
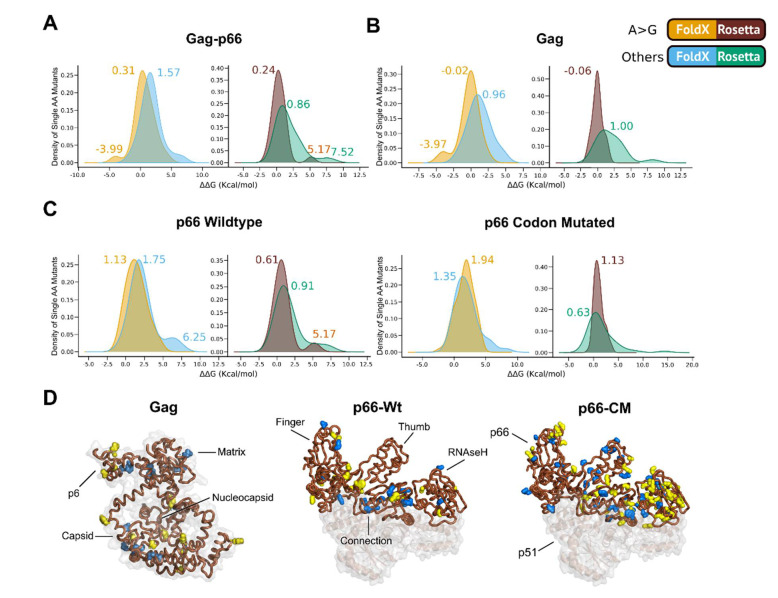
Distributions of the change in mutational free energies (ΔΔG) of pooled mutations. Density plots showing distributions of ΔΔG of all individual amino acid mutations (including hypermutations) from (**A**) Gag-p66, (**B**) Gag (**C**) wild-type RT p66 (p66-Wt) and codon mutated RT p66 (p66-CM) genes. (**D**) Positions of ‘A to G’ mutations in the protein structures of Gag and p66 are shown in yellow while all other mutations are in blue. There were both ‘A to G’ and ‘other’ mutations at position 462 of p66-CM labeled blue. The C and N terminals and locations of major domains and functional protein regions are shown with labels. Peaks are numbered with corresponding ΔΔG values. The mutational free energies were modeled with the Rosetta Cartesian_ddg and FoldX BuildPDB protocols. ΔΔG values or ΔΔG differences between different distributions of >1 kcal for Rosetta (29) and ΔΔG > 0.46 kcal/mol for FoldX (30) were significant. Note that Gag exists in both the compact and extended states and since the former precedes the later during viral assembly, the former was used.

The unique robustness of HIV to resist drugs, immune pressures, and the error prone nature of RTs may be attributed to the ability of viral protein structures to buffer fitness by minimizing the difference in free energy changes or ΔΔG [29]. When mutation(s) caused destabilization (e.g., with ΔΔG > 4 kcal/mol), proteins may adopt a substantially different fold or be misfolded [30]. Our results showed that the distribution of ΔΔG caused by all the mutations in the combined Gag and p66 genes (Gag-p66 in Figure 6A, excluding Protease due to the absence of classified hypermutations) revealed that the majority of the ‘A to G’ mutations did not exert significant effects on the protein stability (FoldX: |ΔΔG| < 0.46 kcal/mol and Rosetta: |ΔΔG| < 1 kcal/mol, shown as yellow and brown peaks in Figure 6A). It is noteworthy in Gag that there were some cases where the ‘A to G’ mutations stabilized the protein (ΔΔG ~ −3.9 kcal/mol, shown as yellow peaks in Figure 6A,B).

On the other hand, the other non ‘A to G’ mutations had more destabilizing effects (Figure 6A, blue and green peaks with |ΔΔG| more or approximate the thresholds for FoldX and Rosetta, respectively). This supports the earlier observation that ‘A to G’ mutations tend to cause little difference in free energy change in the combined Gag-p66 genes, especially since respective secondary peaks were observed in individual distributions of Gag and the wild-type RT p66 mutations (Figure 6B,C).

The results suggest that the HIV-1 genes, particularly RT p66 wild-type, were less sensitive to ‘A to G’ mutations (i.e., ‘A to G’ ΔΔG of 1.13 kcal/mol versus ‘other mutations’ ΔΔG of 1.75 kcal/mol in wild-type p66) as compared to codon mutated p66 as shown as yellow peaks in Figure 6C. This trend was also reflected in the results from Rosetta analysis. 

Thermostability analysis of all the hypermutations showed that the mutations might destabilize Gag and wild-type RT p66 and that the effects were doubled for codon mutated p66 (Supplementary Table S6).

## 3. Discussion

We set out to study the native mutation rates of HIV-1 RT on HIV-1 genes: Gag, protease and RT p66 subunit in a low biosafety single replication cycle model devoid of translational, immune or drug selection pressures. While mutations can be contributed by plasmid hosts (DH5α and/or EXPI293F), Q5 polymerase, and even sequencing artefacts, these were very unlikely given that the mutations/bp/replication for *E. coli* and mammalian cells were estimated at 5.4 × 10^−10^ and 5.0 × 10^−11^, respectively [31], and that Q5 polymerase is one of the most high-fidelity polymerases with an error rate of 5.3 × 10^−7^ sub/base/doubling [32]. Since there may be a role for primer selection bias when priming the first few bases of the genes, the priming areas were excluded from analysis and calculation of mutation rates. The observed biases are thus presumed to be intrinsic to HIV-1 RT and host cell factors. 

While numerous in vitro studies have reported the error rates of HIV-1 RT (as previously reviewed in [26,27]), only two reports utilized HIV-1 gene templates for analysis [33,34]. Our mutation rates of HIV-1 Gag, protease, RT p66, and codon mutated p66 were found to be 4.71 × 10^−5^, 6.03 × 10^−5^, 7.09 × 10^−5^, and 1.88 × 10^−4^ mutations/bp (inclusive of hypermutations), respectively, within the previously reported range of 1.8 × 10^−5^–6.67 × 10^−4^ mutations/bp [26,27]. Across the HIV-1 genes, there was a predominance of transition mutations consistent across most phyla, hypothesized to be for the conservation of protein functions [35,36,37]. Missense mutations occurred at the highest frequency in this study, followed by silent, frameshifts, and nonsense mutations, findings in agreement with the in-built genetic code change probabilities [38].

Different HIV-1 gene templates had varying mutation rates, type of mutations and mutational biases with a general absence of ‘A–T’ or ‘C–G’ mutations in the wild-type HIV genes and were detected only in the codon mutated p66 (see Figure 2) which was 2.7 folds higher than the wild-type HIV-1 RT p66. The RNase H domain of the codon mutated HIV-1 RT p66 had a 7.1 fold increase (3.38 × 10^−4^ mutations/bp) compared to the wild-type (4.79 × 10^−5^ mutations/bp), possibly due to sequence motif protection/susceptibility [28,39]. 

Possible explanations for this may be attributed to the short nucleotide length of protease, thereby avoiding the ‘A to G’ mutational effects elicited by host adenosine deaminases such as double-stranded RNA-specific adenosine deaminase (ADAR). ADAR editing (by either ADAR1 and ADAR2) was suggested to influence cell based in vitro RT-fidelity assays in HIV-1 [40], and mutations in other positive strand RNA viruses [41,42,43]. 

The susceptibility of Gag, p66, and codon mutated p66 to ‘A to G’ hypermutations would result in an accumulation of G bases resulting in a translational bias towards glycine, arginine, valine, and alanine [38]. This will in turn reduce the occurrences of phenylalanine, isoleucine, tyrosine, histidine, and asparagine in these genes. For HIV-1 protease, the mutational bias towards ‘A’ and ‘C’ accumulation, which is also the case when ‘A to G’ hypermutations are not considered for Gag and both p66 genes, would lead to translational bias in the ‘A’ accumulation towards threonine and lysine, isoleucine, asparagine, arginine, and stop codons, while reducing phenylalanine, tryptophan, and cysteine occurrences. Similarly, the accumulation of ‘C’ would lead to biases for proline, serine, leucine, threonine, alanine, and arginine while reducing methionine, lysine, glutamic acid, tryptophan, and more importantly, stop codons. Thus, increasing the mutations rates can push the virus towards lethal mutagenesis, allowing a possible therapeutic intervention to exploit the possible underlying host deaminases e.g., ADAR neighbor preference bases flanking target sites.

Structurally, the ‘A to G’ transitions had less destabilization effects in ΔΔG within Gag and wild-type p66. Gag was found to be less sensitive to the ‘A to G’ mutations than p66 (Rosetta data showed distributions of stabilizing mutations that could have acted as epistatic buffers against destabilizing ones), although this may require confirmation using cleaved Gag models [44]. On the other hand, codon mutated p66 was less resistant to ‘A to G’ changes compared to wild-type p66, showing that there was not just minimization of ΔΔG at the structural level [29] alone, but also at the nucleotide sequence level. With multiple ‘A to G’ substitutions, the average of all p66 mutations (and also Gag) were half that of the codon mutated p66. There were less ‘A to G’ transitions in the wild-type p66 compared to its codon mutated counterpart, showing that the codon usage of the natural wild-type p66 (and possibly Gag) gene protected the genes against the cause of hyper ‘A to G’ substitutions. It is worth noting that due to different empirical thresholds of ΔΔG (in kcal/mol) being considered as “significant” effects between FoldX and Rosetta calculations, the effects were not clear in the use of Rosetta as compared to FoldX. However, similar effect trends were observed in both.

Despite the lack of selection pressure towards functional proteins, there were no mutations in the active sites of the enzymes (protease and p66) nor in the cleavage sites of Gag, with the exception of A431D and F433L in the NC/p1 cleavage site 429RQAN/FLG435 of Gag. The detection of non-cleavage mutations in Gag show that such compensation mutations can occur early in the infection process, even in the absence of drugs. In this experimental model of mimicking a single replication cycle, previously reported clinical drug resistance mutations (see Supplementary Table S5 for Gag, protease, and p66) were detected. In Gag, the rare transient mutation E17K (in the matrix) implicated in cytotoxic T lymphocyte (CTL) immune evasion resistance [45,46]. In protease, K70T, the minority mutation associated with resistance to PIs [47]. In p66, the known polymorphic mutation K103R (in the palm domain), which when combined with V179D (not found in our repertoire), reduced susceptibility to non-nucleoside reverse transcriptase inhibitors (NNRTIs): nevirapine (NVP) and efavirenz (EFV) by about 15 folds [48]. Interestingly, the observed K103R originated from an ‘A to G’ hypermutation event, specifically from “AAA” to “AGA”.

Despite being in lower frequencies, mutations were also found in crucial functional sites. In Gag, P222L occurred in the cyclophilin A (CyPA) binding site (along with G221), potentially affecting the binding of the capsid to CyPA as previously reported for P222A [49]. Given that the probability of P (CCT) to L (CTT) is 0.33 of having a T substitution in the 2nd codon position, and that P (CCT) to A (GCT) is also 0.33 of having the first C mutated to a G, there were equal probabilities. However, when considering that our Gag mutation analysis did not show a transition mutation link between C to G (Figure 2), the clinical mutation is a rare-occurrence or a result of two or more mutation steps. Mutations A431D in the nucleocapsid and P453T in the p6 domain were also observed, with reported clinical drug resistance counterparts as A431V and a L449F/P453T pair [50]. In p66, the substitution F61S implicated in strand synthesis with F61Y/L/W altering activity [51] was present. P95L, in which P95 was reported to be at the dimerization interface for the formation of the bottom of NNRTI pocket [52], and a proposed target amino acid in NNRTI design together with N137 and P140 [53] was also detected. Although P95 was reported to be in a highly conserved location [52], it was present in our limited sample size assay. 

The occurrence of previously reported in vitro and clinical mutations in our assay demonstrate that these mutations can occur very early in infection, even in the absence of selection pressures. The avoidance of inducing mutations at crucial sites at the nucleotide stage and protective effects in natural nucleotide codon usage (compared to p66 codon mutated) to minimize changes in protein ΔΔG, suggests in-built sequence barriers of self-preservation. Given that these mutations occurred within our mimic of a single cycle of replication and that HIV-1 generates approximately 10^9^ virions per day in an infected individual [54], drug resistance virions could have been made within the first day of infection. Evidently, given the limitation of generating exhaustive repertoire of each of the genes, our results are limited in detecting the entire repertoire of possible mutations HIV-1 RT would generate. It is expected that with improved high-throughput sequencing technologies such as next-generation sequencing platforms [55,56] that can allow long reads, it would be possible to detect rare mutations to allow a more comprehensive understanding. Furthermore, such platforms could be applied after adaptations to other RNA viruses and cancer to pre-emptively detect mutations. By studying spontaneous viral receptors mutations, the assay can allow better prediction of viral tropism changes (as evident in current COVID19 spike changes) and for pre-emptive interventions to be designed. However, this would depend also on the mutation rate of the system to be studied, where the lower the mutation rate, the bigger the repertoire sampling required to detect rare mutations.

Given that many HIV proteins can function in intense drug/immune selection environments with significant reduced activity [57,58], a multi-pronged drug intervention against HIV would involve inhibitors against all possible drug resistance mutations. Such inhibitors could include proteins such as the gp120 glycoprotein to disrupt the necessary CD4 interaction and cell entry [59]. Combining such efforts, this could drive HIV towards Muller’s ratchet [60,61,62] by bottlenecking the production of drug resistant functional proteins. The alternative is to augment the mutation rate towards lethal mutagenesis for error catastrophe [63,64] by mutagenic nucleoside analogues [65,66,67]. With significant nonsense mutations or missense mutations that disrupt protein functionality, the replication would then be self-limiting in error-catastrophe.

As it was previously shown that protease and RT drug cross resistance have a structural basis governed by drug resistance mutations [68,69], the bias of restricting specific amino acid changes by the absence of A-T and C-G mutation occurrences can be exploited. However, such an approach will require an in-depth understanding of HIV-RT mutations that are selected for and against at protein functional levels. 

Through such analysis, it is possible to calculate the mutational events leading to the zoonotic transmission of SIV to HIV or that of other viruses better, opening up surveillance of emerging viral threats [2], especially given that RNA viruses are the most likely to species jump [1], which is relevant to the current ongoing COVID19 pandemic.

## 4. Materials and Methods 

### 4.1. Transfection of HIV-1 Gag, Pr and RT Plasmids

HIV-1 Gag was PCR amplified from plasmid p8.91 [70]. HIV-1 Protease (GenBank: AY622223.1), HIV-1 RT p66 (GenBank: K03455.1), and codon mutated p66 genes were gene synthesized (BioBasic Asia Pte Ltd., Singapore). The codon mutated p66 sequence was generated by reverse translation from the amino acid sequence to obtain differing nucleotide sequences while retaining the amino acid sequence (Supplementary Data 1). The genes were then cloned separately into the pTT5 plasmid vector (YouBio), transformed into competent DH5α *E. coli* cells [71], and transfected into EXPI293F cells cultured in Dulbecco’s modified Eagle’s Media supplemented with 10% fetal bovine serum, penicillin/streptomycin, and sodium pyruvate in a 37 °C incubator with CO_2_ supplemented at 5% and transfected (4 × 10^5^ cells/mL) as previously performed [72,73,74]. 

### 4.2. RNA Extraction and cDNA Synthesis

Total RNA from two-day transfected cells with the gene of interest were extracted using TRIzol according to manufacturer’s instructions (Invitrogen, Singapore). cDNA synthesis was performed using recombinant HIV-1 RT subunits: (i) p51 (0.2475 µg) and p66 (0.2125 µg) from Sino Biological Inc, China. (catalogue: 40244-V07E and 40244-V07E1, respectively), (ii) 3 µg DNase-treated RNA, (iii) 5X RT buffer (25 mM Dithiothreitol, 375 mM KCl, 15 mM MgCl_2_, 250 mM Tris-HCl [pH 8.3]), (iv) 50 µM Oligo(dT)_18_ (Thermo Scientific, Singapore), (v) 10 mM dNTP mixture (First Base Pte Ltd., Singapore), and (vi) 40 U RiboLock RNase inhibitor (ThermoFisher Scientific, Singapore), in a single cycle of 25 °C for 18 min, 37 °C for 1 h, and 85 °C for 5 min. RT negative controls were prepared without the addition of HIV-1 RT p51 and p66 subunits. 

### 4.3. Amplification of cDNA and TOPO Cloning

PCR amplification of the cDNA template was performed using the high-fidelity Q5 Polymerase PCR (New England Biolabs, Singapore) with the following in-house adapted primers sets: HIV-1 Gag-F (5′-TAT TAG GAA TTC ATG GGT GCG AGA GCG-3′) and R (5′-CTG GTA AAG CTT CTA GTG GTG GTG GTG-3′); protease-F (5′-GCG GCC GAA TTC ATG CCT CAA ATC AC-3′) and R (5′-TAT AAT AAG CTT CTA GTG GTG GTG GTG-3′); RT p66-F (5′-ATG GCC TTG ACC TTT GCT TTA CTG-3′) and R (5′-CTT GTC GTC ATC GTC TTT GTA GTC–3′); and codon mutated p66-F (5′-GCG GTG ATG GAT GGA CCA AAA GTA AA-3′) and R (5′-CTG CGC CTA ATG ATG ATG ATG AT-3′) as per manufacturer’s recommendations. Oligonucleotide properties were determined using OligoCalc [75]. Thermocycler conditions were set at 98.0 °C (30 s), 35 cycles of 98.0 °C (10 s), 61.3 °C (30 s), and 72.0 °C (1 min 10 s), with a final extension at 72 °C (7 min) for HIV-1 Gag, 98.0 °C (30 s), 35 cycles of 98.0 °C (10 s), 63.0 °C (30 s), and 72.0 °C (45 s), with a final extension at 72 °C (7 min) for protease, 98.0 °C (30 s), 35 cycles of 98.0 °C (10 s), 58.0 °C (30 s), and 72.0 °C (1 min 18 s), with a final extension at 72.0 °C (7 min) for RT p66 and codon mutated p66. PCR products were analyzed by gel electrophoresis with GelApp [76] and purified using the gel extraction and PCR purification kits that were previously described [77]. Purified PCR products were cloned using Zero Blunt TOPO PCR cloning kit (Invitrogen, Singapore) as per the manufacturer’s protocol and transformed into in-house competent DH5α cells as previously described [71]. Transformed DH5α cells were plated and grown overnight at 37 °C on LB agar plates supplemented with kanamycin (50 µg/mL). Transformants were screened using GoTaq PCR (Promega, Singapore) with universal M13 forward and reverse primers prior to sanger sequencing (BioBasic Asia Pte Ltd., Singapore). 

### 4.4. Sequence Analysis 

Sequence assembly and alignment of HIV-1 sequences were performed using the YAQAAT Webserver [78]. DNA2App [79] was used to analyze nucleotide and amino acid sequences. Mutations in the cDNA gene sequences were identified by multiple sequence alignments with characterized HIV-1 sequences from the Los Alamos sequence database [80]. To rule out sequencing artefacts, sequence chromatograms were analyzed. Sequencings were repeated for ambiguous peaks and detected mutations. The mutation rates were calculated as mutations/bp where the total number of mutations and the total nucleotide bases of the respective HIV-1 genes were compared.

A ‘Two Sample logo’ analysis was performed to study the flanking sequences of ‘A to G’ transitions for reported ADAR neighbor sequence preferences [28] that may underlie ‘A to G’ hyper-editing reported for a wide variety of RNA based viruses [41,42,43]. A custom script was written to automate the locating, pooling, and aligning of 4 nucleotides upstream and downstream of all identified adenosine mutants (see Figure 5A for schematic representation). These sequence regions were compared against the respective original sequences using the Two Sample logo software [81] with two sample *t*-test without Bonferroni correction to test for significantly enriched and depleted bases within 9 nucleotides.

### 4.5. In-Silico Assessment on Protein Thermostability Using FoldX and Rosetta Cartesian_ddg 

In silico mutagenesis for Gag was performed on previously modeled compact Gag structures [14] using PyMOL [82] followed by energy minimization using Swiss-PdbViewer [83]. For the RT p66, a HIV-1 RT crystal structure (PDB: 3T19) was used. 

To evaluate protein thermostability, free energy changes with ΔΔG = ΔGmutant-ΔGwild-type were modeled using FoldX5 (Delgado et al., 2019), and Rosetta Cartesian_ddg Version: 2017.52.58848 [84], with ΔΔG < 0 indicating stabilizing and ΔΔG > 0 indicating destabilizing effects. It should be noted that the thresholds of difference in free energy changes, i.e., absolute value of ΔΔG, were used to evaluate the extent of mutational effect on the protein stability, e.g., |ΔΔG| > 0.46 kcal/mol for FoldX 5 (Delgado et al., 2019), and |ΔΔG| > 1.0 kcal/mol for Rosetta energy calculations (Park et al., 2016a). 

Structural models were first relaxed using the FoldX RepairPDB prior to mutagenesis (either with all individual amino acid mutations arising from all ‘A to G’ substitutions or multiple mutations arising from each hypermutation event) using the BuildModel module with default parameters. The process was replicated 10 times (numberOfRuns = 10) and the average ΔG used for comparisons.

For the Rosetta calculations, similar mutagenesis was performed as in the Foldx process. Cartesian-space refinement were performed (1000 replicates) using the ref2015_cart score function to first relax the structures. The lowest scoring model was selected for further calculations. Free energy modeling using the Cartesian_ddg protocol (https://www.rosettacommons.org/docs/latest/cartesian-ddG) with the ref2015_cart score function was used to generate 15 replicates for each mutant structure. The average of the lowest 3 scores were converted into kcal/mol units by multiplying it with the scaling factor α = 0.34 [84].

## 5. Conclusions

In conclusion, we have established an assay and characterized HIV-1 RT mutations on HIV-1 Gag, protease, and RT p66 in a safe non-viral environment, allowing for insights into the mutational bias and mutation rate of HIV in the absence of biological selection pressures. Such assays can provide deeper insights relevant for drug and vaccine development and be applied for horizontal understanding to other viruses with deeper insights into their adaptive trajectories at the sequence and structural levels.

## Figures and Tables

**Table 1:** Calculated error rates of HIV-1 RT on the respective HIV-1 genes.

HIV-1 Gene	No. of Clones	Nucleotide Length	Total No. of Bases	Excluding ‘A to-G’ Hypermutations		Including ‘A to G’ Hypermutations	
				No. of Mutations	Mutation Rate (Mutations/bp)	No. of Mutations	Mutation Rate (Mutations/bp)
Gag	801	1485	1,189,485	40	3.36 × 10^−5^	56	4.71 × 10^−5^
protease	640	285	182,400	11	6.03 × 10^−5^	-	-
RT p66	571	1680	959,280	41	4.27 × 10^−5^	68	7.09 × 10^−5^
Codon mutated RT p66	700	1617	1,131,900	74	6.53 × 10^−5^	213	1.88 × 10^−4^

Mutation rates were calculated as the ratio of the total number of mutations and the total number of nucleotide bases. The primer regions were excluded from the calculations. The rates were within the reported range of 1.8 × 10−5–6.67 × 10−4 as previously reviewed [26,27].

## Data Availability

Data is contained within the article and Supplementary Materials.

## Supplementary Materials

Supplementary Materials can be found at https://www.mdpi.com/1422-0067/22/1/370/s1.
