# Supplementary material for: Spontaneous Mutations in HIV-1 Gag, Protease, RT p66 in the First Replication Cycle and How They Appear: Insights from an In Vitro Assay on Mutation Rates and Types
Source: Int J Mol Sci. 2020 Dec 31;22(1):370. doi: 10.3390/ijms22010370 (PMC7796399; doi:10.3390/ijms22010370)
Supplement: Supplementary file 1 [file ijms-22-00370-s001.pdf]

Supplementary Material

Table S1. HIV-1 Gag nucleotide and amino acid mutations. Free energy values ΔΔG (kcal / mol) were rounded to 2 decimal places. Rows were colour coded based on the type of mutation (Orange = Missense/Non-Conservative mutations, Yellow = Missense/Conservative Mutations Green = Silent mutations, Purple = Frameshift mutations resulting in Insertions, Pink = Frameshift mutations resulting in deletions, Grey = Nonsense mutations).

Variant	Nucleotide Position	Initial Codon	Mutated Codon	Nucleotide Mutation	Amino Acid Position	Initial Amino Acid	Mutated Amino Acid	Amino Acid Mutation	Rosetta Cartesian_ddg ΔΔG (kcal / mol)	FoldX BuildPDB ΔΔG (kcal / mol)	Type of Mutation		Domain
1	49	GAA	AAA	G49A	17	E (Glutamic Acid)	K (Lysine)	E17K	-0.53	-0.19	Missense	Non-conservative	MA
1	759	AGT	AGC	T759C	253	S (Serine)	S (Serine)	-	-	-	Silent		CA
2	574	GGG	AGG	G574A	192	G (Glycine)	R (Arginine)	G192R	-0.79	0.56	Missense	Non-conservative	CA
3	722	AGT	ATT	G722T	241	S (Serine)	I (Isoleucine)	S241I	-1.81	3.00	Missense	Non-conservative	CA
4A,B	1297	TTT	CTT	T1297C	433	F (Phenylalanine)	L (Leucine)	F433L	-0.27	2.98	Missense	Conservative	NC
5	812	AAT	AGT	A812G	271	N (Asparagine)	S (Serine)	N271S	0.99	-0.20	Missense	Conservative	CA
6	1451	TTT	TCT	T1451C	484	F (Phenylalanine)	S (Serine)	F484S	4.65	0.09	Missense	Non-conservative	P6
7	371	CAC	CGC	A371G	124	H (Histidine)	R (Arginine)	H124R	-0.17	0.00	Missense	Conservative	MA
7	869	AAG	AGG	A869G	290	K (Lysine)	R (Arginine)	K290R	-1.53	-0.20	Missense	Conservative	CA
8	1253	AAG	AGG	A1253G	418	K (Lysine)	R (Arginine)	K418R	0.07	-0.36	Missense	Conservative	NC
8	715	ACT	GCT	A715G	239	T (Threonine)	A (Alanine)	T239A	0.26	3.75	Missense	Non-conservative	CA
9A,B	1359	CCA	CCG	A1359G	453	P (Proline)	P (Proline)	-	-	-	Silent		P6
9A,B	605, 606	AAA	AGG	A605G, A606G	202	K (Lysine)	R (Arginine)	K202R	0.06	0.01	Missense	Conservative	CA
9A,B	610	ACC	GCC	A610G	204	T (Threonine)	A (Alanine)	T204A	0.29	1.27	Missense	Non-conservative	CA

9A,B	667	ATT	GTT	A667G	223	I (Isoleucine)	V (Valine)	I223V	-0.11	0.11	Missense	Conservative	CA
9A,B	1386	AGC	AGA	C1386A	462	S (Serine)	R (Arginine)	S462R	1.92	2.86	Missense	Non-conservative	P6
9A,B	640	AGA	GGA	A640G	214	R (Arginine)	G (Glycine)	R214G	1.93	-0.36	Missense	Non-conservative	CA
10	779	-	-	-	-	-	-	-	-	-	Frameshift	Insertion	CA
11A,B	43	CGA	TGA	C43T	15	R (Arginine)	Stop	-	-	-0.20	Nonsense		MA
12	603	TTA	TTG	A603G	201	L (Leucine)	L (Leucine)	-	-	-	Silent		CA
12	606	AAA	AAG	A606G	202	K (Lysine)	K (Lysine)	-	-	-	Silent		CA
12	630	GCA	GCG	A630G	210	A (Alanine)	A (Alanine)	-	-	0.30	Silent		CA
13	964	TTG	CTG	T964C	322	L (Leucine)	L (Leucine)	-	-	-	Silent		CA
14	1116	AAT	AAC	T1116C	372	N (Asparagine)	N (Asparagine)	-	-	1.27	Silent		SP2
15	1281	ACT	ACC	T1281C	427	T (Threonine)	T (Threonine)	-	-	-	Silent		NC
16	665	CCT	CTT	C665T	222	P (Proline)	L (Leucine)	P222L	0.76	0.20	Missense	Conservative	CA
16	124	GAA	AAA	G124A	42	E (Glutamic Acid)	K (Lysine)	E42K	2.4	0.74	Missense	Non-conservative	MA
17A,B	184	GGA	TGA	G184T	62	G (Glycine)	Stop	-	-	2.86	Nonsense		MA
18	316	GAA	TAA	G316T	106	E (Glutamic Acid)	Stop	-	-	-	Nonsense		MA
19A,B	443	TCA	TAA	C443A	148	S (Serine)	Stop	-	-	1.23	Nonsense		CA
20	454	TTA	GTA	T454G	152	L (Leucine)	V (Valine)	L152V	2.7	1.38	Missense	Conservative	CA
21	525	TTA	TTG	A525G	175	L (Leucine)	L (Leucine)	-	-	-	Silent		CA
22A,B	733	GAA	AAA	G733A	245	E (Glutamic Acid)	K (Lysine)	E245K	2.15	-	Missense	Non-conservative	CA
23A,B	834	AGC	AGT	C834T	278	S (Serine)	S (Serine)	-	-	-	Silent		CA
24	921	GAG	GAA	G921A	307	E (Glutamic Acid)	E (Glutamic Acid)	-	-	-	Silent		CA
25	1021	GCG	TCG	G1021T	341	A (Alanine)	S (Serine)	A341S	0.39	-	Missense	Non-conservative	CA
25	1186	GGC	AGC	G1186A	396	G (Glycine)	S (Serine)	G396S	0.95	-	Missense	Non-conservative	NC
26	1050	TGT	TGC	T1050C	350	C (Cystine)	C (Cystine)	-	-	-	Silent		CA
27	1071	GGC	GGT	C1071T	357	G (Glycine)	G (Glycine)	-	-	-	Silent		CA
27	445	CCT	ACT	C445A	149	P (Proline)	T (Threonine)	P149T	0.78	-0.27	Missense	Non-conservative	CA
28	1146	AAT	AAC	T1146C	382	N (Asparagine)	N (Asparagine)	-	-	-	Silent		NC
29	1250	GGA	GTA	G1250T	417	G (Glycine)	V (Valine)	G417V	3.82	0.74	Missense	Conservative	NC

30A,B	1292	GCT	GAT	C1292A	431	A (Alanine)	D (Aspartic Acid)	A431D	0.87	0.73	Missense	Non-conservative	NC
31	1357	CCA	ACA	C1357A	453	P (Proline)	T (Threonine)	P453T	2.15	-	Missense	Non-conservative	P6
32A,B,C,D,E	1360	GAG	TAG	G1360T	454	E (Glutamic Acid)	Stop	-	-	-	Nonsense		P6
33A,B,C	1408	ACA	GCA	A1408G	470	T (Threonine)	A (Alanine)	T470A	-0.84	-	Missense	Non-conservative	P6
33A,B,C	1411	ACT	GCT	A1411G	471	T (Threonine)	A (Alanine)	T471A	-4.03	1.38	Missense	Non-conservative	P6
34	673	CCA	TCA	C673T	225	P (Proline)	S (Serine)	P225S	0.99	1.41	Missense	Non-conservative	CA
35	137	GTT	GCT	T137C	46	V (Valine)	A (Alanine)	V46A	0.77	-	Missense	Conservative	MA
36A,B,C,D,E	673 – 1369	-	-	-	-	-	-	-	-	8.22	Frameshift	Deletion	CA TO P6
37	673 – 1370	-	-	-	-	-	-	-	-	-	Frameshift	Deletion	CA TO P6
38	1497	TCA	TCG	A1497G	499	S (Serine)	S (Serine)	-		-	Silent		P6
38	1499, 1500	CAA	CGG	A1499G, A1500G	500	Q (Glutamine)	R (Arginine)	Q500R	-0.32	-	Missense	Non-conservative	P6
39	578	-	-	-	-	-	-	-	-	0.39	Frameshift	Deletion	CA

Table S2. HIV-1 Protease nucleotide and amino acid mutations. Rows were colour coded based on the type of mutation as in Table S1.

Variant	Nucleotide Position	Initial Codon	Mutated Codon	Nucleotide Mutation	Amino Acid Position	Initial Amino Acid	Mutated Amino Acid	Amino Acid Mutation	Type of Mutation	
1	291	TTA	TTG	A291G	97	L (Leucine)	L (Leucine)	-	Silent	
2	44	GTA	GCA	T44C	15	V (Valine)	A (Alanine)	V15A	Missense	Conservative
3	292	AAT	GAT	A292G	98	N (Asparagine)	D (Aspartic Acid)	N98D	Missense	Non-conservative
4	270	TTG	TTA	G270A	90	L (Leucine)	L (Leucine)	-	Silent	
5	236	CCT	CAT	C236A	79	P (Proline)	H (Histidine)	P79H	Missense	Non-conservative
6	209	AAG	ACG	A209C	70	K (Lysine)	T (Threonine)	K70T	Missense	Non-conservative
7	126	TGG	TGA	G126A	42	W (Tryptophan)	Stop	-	Nonsense	
8	114	TTG	TTA	G114A	38	L (Leucine)	L (Leucine)	-	Silent	
9	136	-	-	-	-	-	-	-	Frameshift	Deletion
10	257 – 286	-	-	-	-	-	-	-	Frameshift	Deletion
11A-E	297	-	-	-	-	-	-	-	Frameshift	Deletion

Table S3. HIV-1 RT p66 nucleotide and amino acid mutations. Free energy values ΔΔG (kcal / mol) were rounded to 2 decimal places. Rows were colour coded based on the type of mutation as in Table S1.

Variant	Nucleotide position	Initial codon	Mutated codon	Nucleotide Mutation	Amino Acid Position	Initial Amino Acid	Mutated Amino Acid	Amino Acid Mutation	Rosetta Cartesian_ddg ΔΔG (kcal / mol)	FoldX BuildPDB ΔΔG (kcal / mol)	Type of mutation		Domain
1	1408	ACT	GCT	A1408G	470	T (Threonine)	A (Alanine)	T470A	2.24	-0.11	Missense	Non-conservative	RNase H
2	561	TTG	TTA	G561A	187	L (Leucine)	L(Leucine)	-			Silent		Palm
3	284	CCA	CTA	C284T	95	P (Proline)	L(Leucine)	P95L	0.87	0.50	Missense	Conservative	Palm
4	322	GTA	ATA	G322A	108	V (Valine)	I (Isoleucine)	V108M	1.84	0.78	Missense	Conservative	Palm
5	73	CCA	ACA	C73A	25	P (Proline)	T(Threonine)	P25T	1.18	1.04	Missense	Non-conservative	Finger
6	1673, 1674	AAA	AAG	A1673G, A1674G	558	K (Lysine)	K (Lysine)	-	-	-	Silent		RNase H
6	1677	GTA	GTG	A1677G	559	V (Valine)	V (Valine)	-	-	-	Silent		RNase H
7	48	ATG	ATT	G48T	16	M (Methionine)	I (Isoleucine)	M16I	1.00	0.15	Missense	Conservative	Finger
8	1585	GAA	TAA	G1585T	529	E (Glutamic acid)	Stop	E529*	-	-	Nonsense		RNase H
9	1123	-	-	-	-	-	-	-	-	-	Frameshift	Insertion	
10	348	TTT	TTC	T348C	116	F (Phenylalanine)	F(Phenylalanine)	-	-	-	Silent		Palm
11	612	GAG	GAT	G612T	204	E (Glutamic acid)	D (Aspartic acid)	E204D	1.74	0.48	Missense	Conservative	Palm

12	1154	-	-	-	-	-	-	-	-	-	Frameshift	Deletion	Connection
13	220	TTA	CTA	T220C	74	L (Leucine)	L(Leucine)	-	-	-	Silent		Finger
14	1653	TTA	TTG	A1653G	551	L (Leucine)	L(Leucine)	-	-	-	Silent		RNase H
15A,B	1661	GCT	GAT	C1661A	554	A (Alanine)	D (Aspartic acid)	A554D	1.91	-0.15	Missense	Non-conservative	RNase H
16	413 – 1451	-	-	-	-	-	-	-	-	-	Frameshift	Deletion	Finger to RNase H
17	1 – 1616	-	-	-	-	-	-	-	-	-	Frameshift	Deletion	Finger to RNase H
18	140 – 1631	-	-	-	-	-	-	-	-	-	Frameshift	Deletion	Finger to RNase H
19	248	GGA	GTA	G248T	83	G (Glycine)	V (Valine)	-	-	-	Silent		Finger
20	104 – 1681	-	-	-	-	-	-	-	-	-	Frameshift	Deletion	Finger to RNase H
21	1258	CCT	ACT	C1258A	420	P (Proline)	T(Threonine)	P420T	2.02	1.68	Missense	Non-conservative	Connection
22	85 – 1616	-	-	-	-	-	-	-	-	-	Frameshift	Deletion	Finger to RNase H
23	717	TGG	TGT	G717T	239	W (Tryptophan)	C (Cysteine)	W239C	5.79	5.20	Missense	Conservative	Thumb
24	345	TAT	TAC	T345C	115	Y (Tyrosine)	Y (Tyrosine)	-	-	-	Silent		Palm
25	683	CTT	CCT	T683C	228	L (Leucine)	P (Proline)	L228P	1.69	1.59	Missense	Conservative	Thumb
26	837	TTA	TTG	A837G	279	L (Leucine)	L(Leucine)	-	-	-	Silent		Thumb
26	969	AAA	AAG	A969G	323	K (Lysine)	K (Lysine)	-	-	-	Silent		Connection
26	975	TTA	TTG	A975G	325	L (Leucine)	L(Leucine)	-	-	-	Silent		Connection
26	976, 978	ATA	GTG	A976G, A978G	326	I (Isoleucine)	V (Valine)	I326V	0.09	0.31	Missense	Conservative	Connection
26	981	GCA	GCG	A981G	327	A (Alanine)	A (Alanine)	-	-	-	Silent		Connection
26	992	AAG	AGG	A992G	331	K (Lysine)	R (Arginine)	K331R	4.20	0.56	Missense	Conservative	Connection
26	1031	GAG	GGG	A1031G	344	E (Glutamic acid)	G (Glycine)	E344G	0.98	0.90	Missense	Non-conservative	Connection
26	1066	AGA	GGA	A1066G	356	R (Arginine)	G (Glycine)	R356G	-0.53	0.66	Missense	Non-conservative	Connection
26	1087, 1088	AAT	GGT	A1087G, A1088G	363	N (Asparagine)	A (Alanine)	N363A	1.85	1.22	Missense	Non-conservative	Connection
26	1096	AAA	GAA	A1096G	366	K (Lysine)	E (Glutamic acid)	K366E	0.30	1.40	Missense	Non-conservative	Connection

26	1125	ATA	ATG	A1125G	375	I (Isoleucine)	M (Methionine)	I375M	1.73	1.27	Missense	Conservative	Connection
27	57 – 1625	-	-	-	-	-	-	-	-	-	Frameshift	Deletion	Finger to RNase H
28	285 – 1488	-	-	-	-	-	-	-	-	-	Frameshift	Deletion	Palm to RNase H
29	1073	AGG	ATG	G1073T	358	R (Arginine)	M (Methionine)	R358G	-0.32	0.70	Missense	Non-conservative	Connection
30	1606	GTA	ATA	G1606A	536	V (Valine)	I (Isoleucine)	V536I	2.41	0.95	Missense	Conservative	RNase H
31	157	GAA	TAA	G157T	53	E (Glutamic acid)	Stop	E53*	-	-	Nonsense		Finger
32	64	AAA	GAA	A64G	22	K (Lysine)	E (Glutamic acid)	K22E	0.95	-0.92	Missense	Non-conservative	Finger
32	237	GAA	GAG	A237G	79	E (Glutamic acid)	E (Glutamic acid)	-	-	-	Silent		Finger
32	267	GAA	GAG	A267G	89	E (Glutamic acid)	E (Glutamic acid)	-	-	-	Silent		Palm
32	308	AAA	AGA	A308G	103	K (Lysine)	R (Arginine)	K103R	-0.09	0.11	Missense	Conservative	Palm
32	406	AAC	GAC	A406G	136	N (Asparagine)	D (Aspartic acid)	N136D	0.95	0.46	Missense	Non-conservative	Finger
33	293	GCA	GTA	C293T	98	A (Alanine)	V (Valine)	A98V	3.72	1.44	Missense	Conservative	Palm
34	1110	GAG	GAT	G1110T	370	E (Glutamic acid)	D (Aspartic acid)	E370D	1.03	1.34	Missense	Conservative	Connection
35	1 – 1579	-	-	-	-	-	-	-	-	-	Frameshift	Deletion	Finger to RNase H
36	861	-	-	-	-	-	-	-	-	-	Frameshift	Deletion	Thumb
37	1140	ATA	ATG	A1140G	380	I (Isoleucine)	M (Methionine)	I380M	2.52	-0.16	Missense	Conservative	Connection
37	1221	CAA	CAG	A1221G	407	Q (Glutamine)	Q (Glutamine)	-	-	-	Silent		Connection
37	1252, 1253	AAT	GGT	A1252G, A1253G	418	N (Asparagine)	G (Glycine)	N418G	1.40	1.01	Missense	Non-conservative	Connection
37	1287	TTA	TTG	A1287G	429	L (Leucine)	L(Leucine)	-	-	-	Silent		RNase H
37	1582	AAG	GAG	A1582G	528	K (Lysine)	E (Glutamic acid)	K528E	3.15	5.17	Missense	Non-conservative	RNase H
38	993	AAG	AAA	G993A	331	K (Lysine)	K (Lysine)	-	-	-	Silent		Connection

39	641	CTT	CCT	T641C	214	L (Leucine)	P (Proline)	L214P	6.94	7.07	Missense	Conservative	Palm
40A,B	996	CAG	CAA	G996A	332	Q (Glutamine)	Q (Glutamine)	-	-	-	Silent		Connection
40A,B	997	GGG	AGG	G997A	333	G (Glycine)	R (Arginine)	G333R	2.41	3.27	Missense	Non-conservative	Connection
40A,B	999	-	-	-	-	-	-	-	-	-	Frameshift	Insertion	Connection
41	111	ATT	ATC	T111C	37	I (Isoleucine)	I (Isoleucine)	-	-	-	Silent		Finger
42	182	TTT	TCT	T182C	61	F (Phenylalanine)	S (Serine)	F61S	1.85	-0.14	Missense	Non-conservative	Finger
42	1553	GTC	GCC	T1553C	518	V (Valine)	A (Alanine)	V518A	2.94	2.35	Missense	Conservative	RNase H

Table S4. Codon mutated HIV-1 RT p66 nucleotide and amino acid mutations. Free energy values ΔΔG (kcal / mol) were rounded to 2 decimal places. Rows were colour coded based on the type of mutation as in Table S1.

Variant	Nucleotide position	Initial codon	Mutated codon	Nucleotide Mutation	Amino Acid Position	Initial Amino Acid	Mutated Amino Acid	Amino Acid Mutation	Rosetta Cartesian_ddg ΔΔG (kcal / mol)	FoldX BuildPDB ΔΔG (kcal / mol)	Type of mutation		Domain
1	1477	GCT	TCT	G1477T	493	A (Alanine)	S (Serine)	A493S	1.66	0.14	Missense	Non-conservative	RNase H
2	199	AAA	GAA	A199G	67	K (Lysine)	E (Glutamic Acid)	K67E	0.36	-0.17	Missense	Non-conservative	Finger
3	1384	ACA	TCA	A1384T	462	T (Threonine)	S (Serine)	T462S	1.30	0.50	Missense	Conservative	RNase H
4A,B	636	TTT	TTC	T636C	212	F (Phenylalanine)	F (Phenylalanine)	-	-	-	Silent		Palm
5A,B	898	CAT	AAT	C898A	300	H (Histidine)	N (Asparagine)	H300N	2.18	-0.01	Missense	Non-conservative	Thumb
6	1198	GAA	AAA	G1198A	400	E (Glutamic Acid)	K (Lysine)	E400K	3.00	-0.20	Missense	Non-conservative	Connection
7	657	TTA	TTG	A657G	219	L (Leucine)	L (Leucine)	-	-	-	Silent		Palm
7	792	TTA	TTG	A792G	264	L (Leucine)	L (Leucine)	-	-	-	Silent		Thumb
7	805	AGA	GGA	A805G	269	R (Arginine)	G (Glycine)	R269G	2.16	0.67	Missense	Non-conservative	Thumb
7	828	GAA	GAG	A828G	276	E (Glutamic Acid)	E (Glutamic Acid)	-	-	-	Silent		Thumb
7	840	TTA	TTG	A840G	280	L (Leucine)	L (Leucine)	-	-	-	Silent		Thumb
7	841	ACA	GCA	A841G	281	T (Threonine)	A (Alanine)	T281A	1.86	0.06	Missense	Non-conservative	Thumb
7	922, 924	AAA	GAG	A922G, A924G	308	K (Lysine)	E (Glutamic Acid)	K308E	3.22	0.74	Missense	Non-conservative	Connection
7	930	TTA	TTG	A930G	310	L (Leucine)	L (Leucine)	-	-	-	Silent		Connection
7	1050	GTA	GTG	A1050G	350	V (Valine)	V (Valine)	-	-	-	Silent		Connection
7	1051	AAA	GAA	A1051G	351	K (Lysine)	E (Glutamic Acid)	K351E	0.30	1.40	Missense	Non-conservative	Connection

8A,B	1165	GAA	TAA	G1165T	389	E (Glutamic Acid)	Stop	E389*	-	-	Nonsense		Connection
9	1501	GAA	AAA	G1501A	501	E (Glutamic Acid)	K (Lysine)	E501K	0.41	0.65	Missense	Non-conservative	RNase H
10	109	CCA	ACA	C109A	37	P (Proline)	T (Threonine)	P37T	2.44	0.70	Missense	Non-conservative	Finger
11	809	GGA	GAA	G809A	270	G (Glycine)	E (Glutamic Acid)	G270E	6.00	3.31	Missense	Non-conservative	Thumb
12	1157	TGG	TTG	G1157T	386	W (Tryptophan)	L (Leucine)	W386L	2.79	2.86	Missense	Conservative	Connection
13	822	TTA	TTT	A822T	274	L (Leucine)	F (Phenylalanine)	L274F	2.11	0.44	Missense	Conservative	Thumb
14	1214	CCA	CAA	C1214A	405	P (Proline)	Q (Glutamine)	P405Q	2.96	1.37	Missense	Non-conservative	Connection
15	89	GGA	GAA	G89A	30	G (Glycine)	E (Glutamic Acid)	G30E	1.06	4.80	Missense	Non-conservative	Finger
16	1180	ACA	GCA	A1180G	394	T (Threonine)	A (Alanine)	T394A	2.00	0.45	Missense	Non-conservative	Connection
17	1150	GAA	TAA	G1150T	384	E (Glutamic Acid)	Stop	E384*	-	-	Nonsense		Connection
18A,B,C	1428	TTA	TTG	A1428G	476	L (Leucine)	L (Leucine)	-	-	-	Silent		RNase H
18A,B,C	1444	ACA	GCA	A1444G	482	T (Threonine)	A (Alanine)	T482A	2.66	1.71	Missense	Non-conservative	RNase H
18A,B,C	1510	AAT	GAT	A1510G	504	N (Asparagine)	D (Aspartic Acid)	N504D	2.49	1.30	Missense	Non-conservative	RNase H
18A,B,C	1519	ATT	GTT	A1519G	507	I (Isoleucine)	V (Valine)	I507V	2.75	1.20	Missense	Conservative	RNase H
18A,B,C	1530	TTA	TTG	A1530G	510	L (Leucine)	L (Leucine)	-	-	-	Silent		RNase H
18A,B,C	1573	AAA	GAA	A1573G	525	K (Lysine)	E (Glutamic Acid)	K525E	1.55	0.58	Missense	Non-conservative	RNase H
18A,B,C	1611	GTA	GTG	A1611G	537	V (Valine)	V (Valine)	-	-	-	Silent		RNase H
18A,B,C	1621	ATT	GTT	A1621G	541	I (Isoleucine)	V (Valine)	I541V	1.17	1.46	Missense	Conservative	RNase H
18A,B,C	1627	AAA	GAA	A1627G	543	K (Lysine)	E (Glutamic Acid)	K543E	-0.99	0.17	Missense	Non-conservative	RNase H
18A,B,C	1635	TTA	TTG	A1635G	545	L (Leucine)	L (Leucine)	-	-	-	Silent		RNase H

19	1444 - 1473	-	-	-	-	-	-	-	-	-	Frameshift	Deletion	RNase H
20	989	CCA	CAA	C989A	330	P (Proline)	Q (Glutamine)	P330Q	0.76	1.20	Missense	Non-conservative	Connection
21	994	AAA	GAA	A994G	332	K (Lysine)	E (Glutamic Acid)	K332E	1.44	0.85	Missense	Non-conservative	Connection
21	1016	TAT	TGT	A1016G	339	Y (Tyrosine)	C (Cysteine)	Y339C	3.58	2.75	Missense	Non-conservative	Connection
21	1050	GTA	GTG	A1050G	350	V (Valine)	V (Valine)	-	-	-	Silent		Connection
21	1053	AAA	AAG	A1053G	351	K (Lysine)	K (Lysine)	-	-	-	Silent		Connection
21	1077	AAA	AAG	A1077G	359	K (Lysine)	K (Lysine)	-	-	-	Silent		Connection
21	1099	ATT	GTT	A1099G	367	I (Isoleucine)	V (Valine)	I367V	1.98	0.97	Missense	Conservative	Connection
21	1123	AAA	GAA	A1123G	375	K (Lysine)	E (Glutamic Acid)	K375E	-0.24	3.15	Missense	Non-conservative	Connection
21	1139, 1140	AAA	AGG	A1139G, A1140G	380	K (Lysine)	R (Arginine)	K380R	-0.04	0.53	Missense	Conservative	Connection
21	1169	TAT	TGT	A1169G	390	Y (Tyrosine)	C (Cysteine)	Y390C	2.52	2.50	Missense	Non-conservative	Connection
21	1180, 1182	ACA	GCG	A1180G, A1182G	394	T (Threonine)	A (Alanine)	T394A	2.00	0.45	Missense	Non-conservative	Connection
21	1221	TTA	TTG	A1221G	407	L (Leucine)	L (Leucine)	-	-	-	Silent		Connection
21	1242	TTA	TTG	A1242G	414	L (Leucine)	L (Leucine)	-	-	-	Silent		RNase H
21	1277	TAT	TGT	A1277G	426	Y (Tyrosine)	C (Cysteine)	Y426C	4.02	2.42	Missense	Non-conservative	RNase H
21	1384	ACA	GCA	A1384G	462	T (Threonine)	A (Alanine)	T462A	-0.17	-0.95	Missense	Non-conservative	RNase H
21	1392	TTA	TTG	A1392G	464	L (Leucine)	L (Leucine)	-	-	-	Silent		RNase H
21	1399	ATT	GTT	A1399G	467	I (Isoleucine)	V (Valine)	I467V	1.64	0.94	Missense	Conservative	RNase H
21	1407	TTA	TTG	A1407G	469	L (Leucine)	L (Leucine)	-	-	-	Silent		RNase H
21	1434	GTA	GTG	A1434G	478	V (Valine)	V (Valine)	-	-	-	Silent		RNase H

21	1435	AAT	GAT	A1435G	479	N (Asparagine)	D (Aspartic Acid)	N479D	1.32	2.63	Missense	Non-conservative	RNase H
21	1464	TTA	TTG	A1464G	488	L (Leucine)	L (Leucine)	-	-	-	Silent		RNase H
21	1530	TTA	TTG	A1530G	510	L (Leucine)	L (Leucine)	-	-	-	Silent		RNase H
21	1531	ATT	GTT	A1531G	511	I (Isoleucine)	V (Valine)	I511V	1.50	0.81	Missense	Conservative	RNase H
22	88	GGA	TGA	G88T	30	G (Glycine)	Stop	G30*	-	-	Nonsense		Finger
23	1474	CAA	AAA	C1474A	492	Q (Glutamine)	K (Lysine)	Q492K	0.49	-0.65	Missense	Non-conservative	RNase H
24	153	-	-	-	-	-	-	-	-	-	Frameshift	Insertion	
25	1232	TGG	TTG	G1232T	411	W (Tryptophan)	L (Leucine)	W411L	0.60	-0.15	Missense	Conservative	Connectio n
26	1063	GAA	TAA	G1063T	355	E (Glutamic Acid)	Stop	E355*	-	-	Nonsense		Connectio n
27	800	CTT	CCT	T800C	267	L (Leucine)	P (Proline)	L267P	8.25	5.17	Missense	Conservative	Thumb
28	568	TTA	CTA	T568C	190	L (Leucine)	L (Leucine)	-	-	-	Silent		Palm
29	1396	GCA	TCA	G1396T	466	A (Alanine)	S (Serine)	A466S	2.66	0.98	Missense	Non-conservative	RNase H
30	723	GAT	GAC	T723C	241	D (Aspartic Acid)	D (Aspartic Acid)	-	-	-	Silent		Thumb
31	682	CCA	TCA	C682T	228	P (Proline)	S (Serine)	P228S	2.32	1.56	Missense	Non-conservative	Thumb
32	21	AAA	AAG	A21G	7	K (Lysine)	K (Lysine)	-	-	-	Silent		Finger
32	33	TTA	TTG	A33G	11	L (Leucine)	L (Leucine)	-	-	-	Silent		Finger
32	39	GAA	GAG	A39G	13	E (Glutamic Acid)	E (Glutamic Acid)	-	-	-	Silent		Finger
32	44	AAA	AGA	A44G	15	K (Lysine)	R (Arginine)	K15R	0.84	-0.01	Missense	Conservative	Finger
32	57	TTA	TTG	A57G	19	L (Leucine)	L (Leucine)	-	-	-	Silent		Finger
32	70	ACA	GCA	A70G	24	T (Threonine)	A (Alanine)	T24A	1.51	-0.13	Missense	Non-conservative	Finger

32	84	AAA	AAG	A84G	28	K (Lysine)	K (Lysine)	-	-	-	Silent		Finger
32	100, 102	AAA	GAG	A100G, A102G	34	K (Lysine)	E (Glutamic Acid)	K34E	3.11	1.24	Missense	Non-conservative	Finger
32	142	ATT	GTT	A142G	48	I (Isoleucine)	V (Valine)	I48V	1.75	0.68	Missense	Conservative	Finger
32	146	AAG	AGG	A146G	49	K (Lysine)	R (Arginine)	K49R	-2.23	0.22	Missense	Conservative	Finger
32	151, 153	AAA	GAG	A151G, A153G	51	K (Lysine)	E (Glutamic Acid)	K51E	3.38	-0.08	Missense	Non-conservative	Finger
32	165	AAA	AAG	A165G	55	K (Lysine)	K (Lysine)	-	-	-	Silent		Finger
32	177	TTA	TTG	A177G	59	L (Leucine)	L (Leucine)	-	-	-	Silent		Finger
32	364	AAT	GAT	A364G	122	N (Asparagine)	D (Aspartic Acid)	N122D	-0.47	0.52	Missense	Non-conservative	Finger
33	225	GTA	GTG	A225G	75	V (Valine)	V (Valine)	-	-	-	Silent	-	Palm
33	784	CGT	AGT	C784A	262	R (Arginine)	S (Serine)	R262S	1.20	2.08	Missense	Non-conservative	Thumb
34	1010	GGA	GTA	G1010T	337	G (Glycine)	V (Valine)	G337V	2.49	14.36	Missense	Conservative	Connection
35	377 – 401	-	-	-	-	-	-	-	-	-	Frameshift	Deletion	Finger
36	1127	CTT	CCT	T1127C	376	L (Leucine)	P (Proline)	L376P	5.83	8.53	Missense	Conservative	Connection
37A,B	460	GAA	AAA	G460A	154	E (Glutamic Acid)	K (Lysine)	E154K	1.54	-0.19	Missense	Non-conservative	Palm
38	1429	GAA	AAA	G1429A	477	E (Glutamic Acid)	K (Lysine)	E477K	1.13	-0.75	Missense	Non-conservative	RNase H
39	1573	AAA	GAA	A1573G	525	K (Lysine)	E (Glutamic Acid)	K525E	1.55	0.58	Missense	Non-conservative	RNase H
39	1579	ATT	GTT	A1579G	527	I (Isoleucine)	V (Valine)	I527V	-0.26	0.63	Missense	Conservative	RNase H
39	1588	AAT	GAT	A1588G	530	N (Asparagine)	D (Aspartic Acid)	N530D	1.33	1.47	Missense	Non-conservative	RNase H
39	1596	CAA	CAG	A1596G	532	Q (Glutamine)	Q (Glutamine)	-	-	-	Silent		RNase H
39	1599	GTA	GTG	A1599G	533	V (Valine)	V (Valine)	-	-	-	Silent		RNase H

39	1604	AAA	AGA	A1604G	535	K (Lysine)	R (Arginine)	K535R	1.04	0.05	Missense	Conservative	RNase H
39	1624	AGA	GGA	A1624G	542	R (Arginine)	G (Glycine)	R542G	0.75	0.80	Missense	Non-conservative	RNase H
39	1632	GTA	GTG	A1632G	544	V (Valine)	V (Valine)	-	-	-	Silent		RNase H
39	1635	TTA	TTG	A1635G	545	L (Leucine)	L (Leucine)	-	-	-	Silent		RNase H
40	1069	GTT	ATT	G1069A	357	V (Valine)	I (Isoleucine)	V357I	1.18	-0.95	Missense	Conservative	Connection
41	634	TTT	ATT	T634A	212	F (Phenylalanine)	I (Isoleucine)	F212I	5.04	3.25	Missense	Conservative	Palm
42	1242	TTA	TTG	A1242G	414	L (Leucine)	L (Leucine)	-	-	-	Silent		RNase H
42	1248	AAA	AAG	A1248G	416	K (Lysine)	K (Lysine)	-	-	-	Silent		RNase H
42	1260	GTA	GTG	A1260G	420	V (Valine)	V (Valine)	-	-	-	Silent		RNase H
42	1272	ACA	ACG	A1272G	424	T (Threonine)	T (Threonine)	-	-	-	Silent		RNase H
42	1277	TAT	TGT	A1277G	426	Y (Tyrosine)	C (Cysteine)	Y426C	4.02	2.42	Missense	Non-conservative	RNase H
42	1302	GAA	GAG	A1302G	434	E (Glutamic Acid)	E (Glutamic Acid)	-	-	-	Silent		RNase H
42	1307	AAA	AGA	A1307G	436	K (Lysine)	R (Arginine)	K436R	0.06	0.27	Missense	Conservative	RNase H
42	1342	AGA	GGA	A1342G	448	R (Arginine)	G (Glycine)	R448G	3.91	2.31	Missense	Non-conservative	RNase H
42	1349, 1350	AAA	AGG	A1349G, A1350G	450	K (Lysine)	R (Arginine)	K450R	1.75	0.37	Missense	Conservative	RNase H
42	1353	GTA	GTG	A1353G	451	V (Valine)	V (Valine)	-	-	-	Silent		RNase H
42	1381, 1382, 1383	AAA	GGG	A1381G, A1382G, A1383G	461	K (Lysine)	G (Glycine)	K461G	2.08	1.55	Missense	Non-conservative	RNase H
42	1399	ATT	GTT	A1399G	467	I (Isoleucine)	V (Valine)	I467V	1.64	0.94	Missense	Conservative	RNase H
42	1428	TTA	TTG	A1428G	476	L (Leucine)	L (Leucine)	-	-	-	Silent		RNase H

42	1444	ACA	GCA	A1444G	482	T (Threonine)	A (Alanine)	T482A	2.66	1.71	Missense	Non-conservative	RNase H
42	1450	AGT	GGT	A1450G	484	S (Serine)	G (Glycine)	S484G	3.32	0.86	Missense	Non-conservative	RNase H
42	1464	TTA	TTG	A1464G	488	L (Leucine)	L (Leucine)	-	-	-	Silent		RNase H
42	1471	ATT	GTT	A1471G	491	I (Isoleucine)	V (Valine)	I491V	2.31	1.14	Missense	Conservative	RNase H
42	1476	CAA	CAG	A1476G	492	Q (Glutamine)	Q (Glutamine)	-	-	-	Silent		RNase H
42	1482	CAA	CAG	A1482G	494	Q (Glutamine)	Q (Glutamine)	-	-	-	Silent		RNase H
42	1510	AAT	GAT	A1510G	504	N (Asparagine)	D (Aspartic Acid)	N504D	2.49	1.30	Missense	Non-conservative	RNase H
42	1534, 1535	AAG	GGG	A1534G, A1535G	512	K (Lysine)	G (Glycine)	K512G	0.74	0.21	Missense	Non-conservative	RNase H
42	1542	GAA	GAG	A1542G	514	E (Glutamic Acid)	E (Glutamic Acid)	-	-	-	Silent		RNase H
42	1543	AAA	GAA	A1543G	515	K (Lysine)	E (Glutamic Acid)	K515E	3.35	2.49	Missense	Non-conservative	RNase H
42	1554	TTA	TTG	A1554G	518	L (Leucine)	L (Leucine)	-	-	-	Silent		RNase H
42	1573, 1574	AAA	GGA	A1573G, A1574G	525	K (Lysine)	G (Glycine)	K525G	1.91	1.20	Missense	Non-conservative	RNase H
42	1588	AAT	GAT	A1588G	530	N (Asparagine)	D (Aspartic Acid)	N530D	1.33	1.47	Missense	Non-conservative	RNase H
42	1596	CAA	CAG	A1596G	532	Q (Glutamine)	Q (Glutamine)	-	-	-	Silent		RNase H
42	1599	GTA	GTG	A1599G	533	V (Valine)	V (Valine)	-	-	-	Silent		RNase H
42	1612	AGT	GGT	A1612G	538	S (Serine)	G (Glycine)	S538G	0.78	0.23	Missense	Non-conservative	RNase H
42	1627	AAA	GAA	A1627G	543	K (Lysine)	E (Glutamic Acid)	K543E	-0.99	0.17	Missense	Non-conservative	RNase H
42	1635	TTA	TTG	A1635G	545	L (Leucine)	L (Leucine)	-	-	-	Silent		RNase H
43	829	GTA	ATA	G829A	277	V (Valine)	I (Isoleucine)	V277I	-0.02	-0.31	Missense	Conservative	Thumb
44	1073	-	-	-	-	-	-	-	-	-	Frameshift	Deletion	Connection

45	573	CGT	CGC	T573C	191	R (Arginine)	R (Arginine)	-	-	-	Silent		Palm
46	139	GCT	ACT	G139A	47	A (Alanine)	T (Threonine)	A47T	1.73	0.83	Missense	Non-conservative	Finger
47A,B,C	981	TAT	TAC	T981C	327	Y (Tyrosine)	Y (Tyrosine)	-	-	-	Silent		Connection
48	1030	GGT	TGT	G1030T	344	G (Glycine)	C (Cysteine)	G344C	1.51	3.74	Missense	Conservative	Connection
48	1267	GAA	TAA	G1267T	423	E (Glutamic Acid)	Stop	E423*	-	-	Nonsense		RNase H
49	889	GAA	AAA	G889A	297	E (Glutamic Acid)	K (Lysine)	E297K	0.34	-0.08	Missense	Non-conservative	Thumb
50	1002	TTA	TTG	A1002G	334	L (Leucine)	L (Leucine)	-	-	-	Silent		Connection
50	1005	AAA	AAG	A1005G	335	K (Lysine)	K (Lysine)	-	-	-	Silent		Connection
50	1006	ACA	GCA	A1006G	336	T (Threonine)	A (Alanine)	T336A	-0.18	1.50	Missense	Non-conservative	Thumb
50	1013, 1014	AAA	AGG	A1013G, A1014G	338	K (Lysine)	R (Arginine)	K338R	1.88	0.45	Missense	Conservative	Thumb
50	1024	ATG	GTG	A1024G	342	M (Methionine)	V (Valine)	M342V	2.15	1.04	Missense	Conservative	Thumb
50	1059	TTA	TTG	A1059G	353	L (Leucine)	L (Leucine)	-	-	-	Silent		Connection
50	1247, 1248	AAA	AGG	A1247G, A1248G	416	K (Lysine)	R (Arginine)	K416R	0.54	0.05	Missense	Conservative	RNase H
50	121428	TTA	TTG	A1428G	476	L (Leucine)	L (Leucine)	-	-	-	Silent	-	RNase H
50	1431	GAA	GAG	A1431G	477	E (Glutamic Acid)	E (Glutamic Acid)	-	-	-	Silent	-	RNase H
50	1464	TTA	TTG	A1464G	488	L (Leucine)	L (Leucine)	-	-	-	Silent	-	RNase H
50	1471	ATT	GTT	A1471G	491	I (Isoleucine)	V (Valine)	I491V	2.31	1.14	Missense	Conservative	RNase H
50	1476	CAA	CAG	A1476G	492	Q (Glutamine)	Q (Glutamine)	-	-	-	Silent	-	RNase H
51	1139, 1140	AAA	AGG	A1139G, A1140G	380	K (Lysine)	R (Arginine)	K380R	-0.04	0.53	Missense	Conservative	Connection
52	247	GCA	TCA	G247T	83	A (Alanine)	S (Serine)	A83S	3.19	1.26	Missense	Non-conservative	Palm

53	892	CCA	TCA	C892T	298	P (Proline)	S (Serine)	P298S	-0.38	0.88	Missense	Non-conservative	Thumb
54	1 – 233	-	-	-	-	-	-	-	-	-	Frameshift	Deletion	Finger to Palm
55	1 – 959	-	-	-	-	-	-	-	-	-	Frameshift	Deletion	Finger to Connection
56	1464	TTA	TTG	A1464G	488	L (Leucine)	L (Leucine)	-	-	-	Silent	-	RNase H
57	893	CCA	CTA	C893T	298	P (Proline)	L (Leucine)	P298L	1.38	0.53	Missense	Conservative	Thumb
58	185	TTT	TCT	T185C	62	F (Phenylalanine)	S (Serine)	F62S	3.73	3.96	Missense	Non-conservative	Finger
59	1143	GAA	GAG	A1143G	381	E (Glutamic Acid)	E (Glutamic Acid)	-	-	-	Silent	-	Connection
60	1447	GAT	TAT	G1447T	483	D (Aspartic Acid)	Y (Tyrosine)	D483Y	-1.02	-1.91	Missense	Non-conservative	RNase H
61A,B	1221	TTA	TTG	A1221G	407	L (Leucine)	L (Leucine)	-	-	-	Silent	-	Connection
61A,B	1225	AAA	GAA	A1225G	409	K (Lysine)	E (Glutamic Acid)	K409E	2.45	0.09	Missense	Non-conservative	Connection
61A,B	1260	GTA	GTG	A1260G	420	V (Valine)	V (Valine)	-	-	-	Silent	-	RNase H
61A,B	1263	GGA	GGG	A1263G	421	G (Glycine)	G (Glycine)	-	-	-	Silent	-	RNase H
61A,B	1297	AGA	GGA	A1297G	433	R (Arginine)	G (Glycine)	R433G	-0.78	0.18	Missense	Non-conservative	RNase H
61A,B	1308	AAA	AAG	A1308G	436	K (Lysine)	K (Lysine)	-	-	-	Silent		RNase H
62	1513	CAA	AAA	C1513A	505	Q (Glutamine)	K (Lysine)	Q505K	0.04	0.07	Missense	Non-conservative	RNase H
63	970	TAT	GAT	T970G	324	Y (Tyrosine)	D (Aspartic Acid)	Y324D	3.80	6.55	Missense	Non-conservative	Connection
64	167	TGG	TTG	G167T	56	W (Tryptophan)	L (Leucine)	W56L	2.82	1.21	Missense	Conservative	Finger
65	821	TTA	TCA	T821C	274	L (Leucine)	S (Serine)	L274S	0.34	1.65	Missense	Non-conservative	Thumb
66	1172	TGG	TTG	G1172T	391	W (Tryptophan)	L (Leucine)	W391L	0.77	-0.15	Missense	Conservative	Connection

67	114	GAA	GAG	A114G	38	E (Glutamic Acid)	E (Glutamic Acid)	-	-	-	Silent	-	Finger
67	135	GTA	GTG	A135G	45	V (Valine)	V (Valine)	-	-	-	Silent	-	Finger
67	145, 146	AAG	GGG	A145G, A146G	49	K (Lysine)	G (Glycine)	K49G	4.06	1.06	Missense	Non-conservative	Finger
68	895	GTA	ATA	G895A	299	V (Valine)	I (Isoleucine)	V299I	0.03	-0.03	Missense	Conservative	Thumb
69	371	ACA	AAA	C371A	124	T (Threonine)	K (Lysine)	T124K	1.70	-0.85	Missense	Non-conservative	Finger
70	772	GGT	AGT	G772A	258	G (Glycine)	S (Serine)	G258S	2.20	3.78	Missense	Non-conservative	Thumb
71	1247, 1248	AAA	AGG	A1247G, A1248G	416	K (Lysine)	R (Arginine)	K416R	0.54	0.05	Missense	Conservative	RNase H
71	1325	TAT	TGT	A1325G	442	Y (Tyrosine)	C (Cysteine)	Y442C	3.52	3.54	Missense	Non-conservative	RNase H
71	1330	ACA	GCA	A1330G	444	T (Threonine)	A (Alanine)	T444A	2.34	3.61	Missense	Non-conservative	RNase H
71	1403	TAT	TGT	A1403G	468	Y (Tyrosine)	C (Cysteine)	Y468C	3.25	1.94	Missense	Non-conservative	RNase H
72	137 – 168	-	-	-	-	-	-	-	-	-	Frameshift	Deletion	Finger
73	436	CAA	TAA	C436T	146	Q (Glutamine)	Stop	Q146*	-	-	Nonsense	-	Palm
74	1016	TAT	TGT	A1016G	339	Y (Tyrosine)	C (Cysteine)	Y339C	3.58	2.75	Missense	Non-conservative	Connectio n
74	1065	GAA	GAG	A1065G	355	E (Glutamic Acid)	E (Glutamic Acid)	-	-	-	Silent	-	Connectio n
75	587	AGA	ATA	G587T	196	R (Arginine)	I (Isoleucine)	R196I	0.35	0.03	Missense	Non-conservative	Palm
76	998	AAT	AGT	A998G	333	N (Asparagine)	S (Serine)	N333S	2.15	1.03	Missense	Conservative	Connectio n
77	1444	ACA	GCA	A1444G	482	T (Threonine)	A (Alanine)	T482A	2.66	1.71	Missense	Non-conservative	RNase H
78	666	GAT	GAC	T666C	222	N (Asparagine)	N (Asparagine)	-	-	-	Silent	-	Palm
79	882	ATT	ATC	T882C	294	I (Isoleucine)	I (Isoleucine)	-	-	-	Silent	-	Thumb
80	167	TGG	TAG	G167A	56	W (Tryptophan)	Stop	W56*	-	-	Nonsense	-	Finger

81	710	TGG	TTG	G710T	237	W (Tryptophan)	L (Leucine)	W237L	4.80	0.48	Missense	Conservative	Thumb
82	876	CGT	CGC	T876C	292	R (Arginine)	R (Arginine)	-	-	-	Silent	-	Thumb
83	1219	TTA	CTA	T1219C	407	L (Leucine)	L (Leucine)	-	-	-	Silent	-	Connection
84A,B	694 - 717	-	-	-	-	-	-	-	-	-	-	Deletion	Thumb

Table S5. Clinical, drug resistant and mutations with reported functions of HIV-1 Gag, Protease and RT p66.

Mutation	Domain	Implication / Reported Function	Type	Reference
**HIV-1 Gag**				
E17K	Matrix	CTL immune evasion resistance.<br>Rare and transient mutation.	Clinical Isolate; In Vitro	[1,2]
		E17K was acquired when selecting with novel PI GRL-0519, along with V84A, G61E and D152N	In Vitro	[3]
E42K	Matrix	Compensatory mutations (E42K and P10L) responsible for enhanced infectivity, to overcome deletion in stem-loop 1	In vivo	[4]
G192R	Capsid	G192W (denoted as G60W) increased the number of viral particles at N-terminal domain of capsid protein	In vitro	[5]
K202R	Capsid	Part of PF74 (capsid inhibitor) binding site with Q67H and T107N.<br>Conferred low-level resistance to capsid inhibitor (PF74), impaired HIV-1 infectivity by 90% and reduced PF74 binding to HIV-1 particles	In vitro	[6]
R214G	Capsid	Alanine scanning revealed 3-fold decrease in infectivity	In vitro	[7]
P222L	Capsid	Cyclophilin A (CyPA) binding site (along with G221), with mutant P222A found to disrupt binding to CyPA.	In vitro	[8]
I223V	Capsid	CTL immune evasion resistance.<br>Known compensatory mutation for T242N escape mutation.	In vitro	[9,10]
S241I	Capsid	Transiently observed variant (TiTLQEQIGW) with no reported function	Clinical Isolate	[11]
E245K	Capsid	E245D found to have diminished IFN-γ response	In vivo	[12]
N271S	Capsid	CTL immune evasion resistance.<br>Known rare and transient mutation.	In vitro	[13]
K290R	Capsid	Did not respond to inositol hexakisphosphate (IP6) and s-CANC, consistent with high degree of lysine conservation	In vitro	[14]
A431D	Nucleocapsid	Amino acid position 431 found to be influenced by positive selection.<br>A431V found in patient after PI treatment.	Clinical Isolate	[15]
		Reduced susceptibility to ritonavir by 3.8-fold	In vitro	[16]
P453T	P6	Amino acid 453 found to be influenced by positive selection.<br>P453L found in patient after PI treatment.	Clinical Isolate	[15]
		L449F/P453T were selected after high-pressure passage with protease inhibitor GW640385	In vitro	[17]
T470A	P6	Associated with reduced replication capacity, with polymorphism increasing over course of epidemic in Japan	Clinical Isolate	[18]
		Indicated as an escape variant, peptide titration using PBMCs of HLA-Cw*08 patient demonstrated that peptide containing T470A was more weakly recognised than wild-type	In vivo	[19]
**HIV-1 Protease**				
N98D	-	N98I was observed when performing transposon-directed base-exchange mutagenesis using a random mutant library, function unknown	In vitro	[20]
K70T	-	Minority mutation (2%) associated with resistance to protease inhibitors	Clinical Isolate	[21]
**HIV-1 RT p66**				
F61S	Finger	F61 plays an important role in strand displacement synthesis, with F61Y and F61L increasing efficiency and reduced processivity, while F61W reducing activity	In vitro	[22]
P95L	Palm	P95 is a highly conserved location that makes up important dimerization interface that contributes to formation of bottom of NNRTI pocket	Clinical isolate	[23]
		P95 is a proposed target amino acid in design of novel NNRTIs or dimerization (when together with N137 and P140)	In vitro	[24]

A98V	Palm	A98 is a NNRTI-associated mutation (5.2%)	Clinical isolate	[25]
		A98G confers low-level (~2-fold) resistance to NVP with uncertain virological effects which rarely occurs in drug-naive patients.<br>A98S is a common polymorphism not associated with NNRTI resistance.	Clinical isolate	[26]
		A98G is associated with etravirine resistance, polymorphic in non-B subtypes	Clinical isolate	[27,28]
		A98S is present at low variability in drug-naïve patient (6.8%)	Clinical isolate	[23]
		A98G is selected by nevirapine (NVP)	Clinical Isolate; In vitro	[29]
K103R	Palm	No changes in NNRTI susceptibility alone. Has synergistic effect on NNRTI when combined with V179D	Clinical Isolate; In vitro	[30]
V108M	Palm	V108 is a NNRTI-associated mutation (15.2%)	Clinical Isolate	[31]
		V108I shown to indirectly confer resistance via alterations of drug stacking interactions of the drug through Y181	Crystallog raphy	[32]
N136D	Finger	N136 is essential to preserve catalytic activity, resulting in increased amounts of free p51 and p66 monomers. Mutant N136D decreased inhibitory activity of NNRTI by 1.4- to 6-fold.	In vitro	[33]
E204D	Palm	E204D and E204K are polymorphisms observed in drug naïve patients	Clinical Isolate	[34]
L214P	Palm	L214K found in 6 patients who failed rilpivirine-containing ART	Clinical Isolate	[35]
L228P	Thumb	L228I confers low-level resistance to etravirine, and in combination with Y188C, displays high level of cross resistance to NVP and EFV.	In vitro	[36]
		L228H/R strongly associated with NRTI therapy, L228N is an undifferentiated RTI-selected mutation	Clinical Isolate	[37]
		L228 substitutions strongly associated with TAMs in treated patients	Clinical Isolate	[38]
		L228Q associated with NVP resistance in NRTI-exposed, NNRTI-naive subjects	Clinical Isolate; In vitro	[39]
		L228H/M/R is a polymorphism associated with reduced virological response to didanosine (ddI)	Clinical Isolate	[40]
		L228 associated with patients receiving multiple nucleoside analog inhibitors	Clinical Isolate	[41]
		L228H/R involved in regulation of resistance to NNRTIs	Clinical Isolate	[42]
W239C	Thumb	W239 interacts through P-P interactions with Y318, involved in resistance to NVP and DLV	Clinical Isolate; In vitro	[43]
I326V	Connection	Decreased proportion in NRTI-treated patients when compared to treatment-naive	Clinical Isolate	[44]
K331R	Connection	K331A impairs RT dimerization	In vitro	[45]
G333R	Connection	G333D/E is critical in facilitating dual resistance of AZT and 3TC resistance mutation	In vitro	[46]
N363A	Connection	Mutation in p51 subunit reduces ability to associate with p66 unit	In vitro	[47]
K366E	Connection	K366R is selected in NRTI-treated subjects	Clinical Isolate	[44]
T470A	RNase H	Frequency of T470N decreased in treatment-experienced subtype B isolates compared to drug-naïve isolates	Clinical Isolate	[44]

		T470A/N/G/R found in both naïve and pre-treated patients, T470P/S/E/K mutated more frequently in pre-treated patients	Clinical Isolate	[48]
V536I	RNase H	Polymorphism	Clinical Isolate	[49]
A554D	RNase H	A554T/L/K was found to mutate more frequently in pre-treated than in naive patients, suggesting role in NRTI resistance	Clinical Isolate	[48]

Table S6. Change in mutational free energies (ΔΔG, kcal/mol) of experimentally generated multiple mutation variants.

Gene	Amino acid multiple mutation	ΔΔG (kcal / mol)		Average ΔΔG (kcal / mol)	
		Rosetta Cartesian_ ddg	FoldX BuildPDB	Rosetta Cartesian_ ddg	FoldX BuildPDB
Gag	K202R, T204A, I223V, S462R, R214G	2.29	5.65	0.37	1.75
	E42K, P222L	1.92	0.51		
	A341S, G396S	1.61	3.31		
	T470A, T471A	-4.36	-2.46		
p66 Wildtype	I326V, K331R, E341G, R353G, N360A, K363E, I375M	0.80	5.09	2.53	3.25
	K22E, K103R, N136D	-0.56	-0.37		
	I380M, N418G, K528E	6.17	6.04		
	F61S, V518A	3.69	2.24		
p66 Codon Mutated	R269, T281A, K308E, K351E	2.63	3.01	6.48	7.00
	T482A, N504D, I507V, K543E, I541V, K543E	5.81	6.07		
	T482A, N504D, I507V, K525E, I541V, K543E, K332E, Y339C, I367V, K375E, K380R, Y390C, T394A, Y426C, T462A, I467V, N479D, I511V	12.35	16.40		
	K15R, T24A, K34E, I48V, K49R, K51E, N122D	6.79	1.86		
	K525E, I527V, N530D, K535R, R542G	3.09	4.34		
	Y426C, K436R, R448G, K450R, K461G, I467V, T482A, S484G, I491V, N504D, K512G, K515E, K525G, N530D, S538G, K543E	17.19	18.68		
	T336A, K338R, M342V, K416R, I491V	0.65	2.87		
	K409E, R433G	0.37	0.24		
	K416R, Y442C, T444A, Y468C	9.41	9.48		

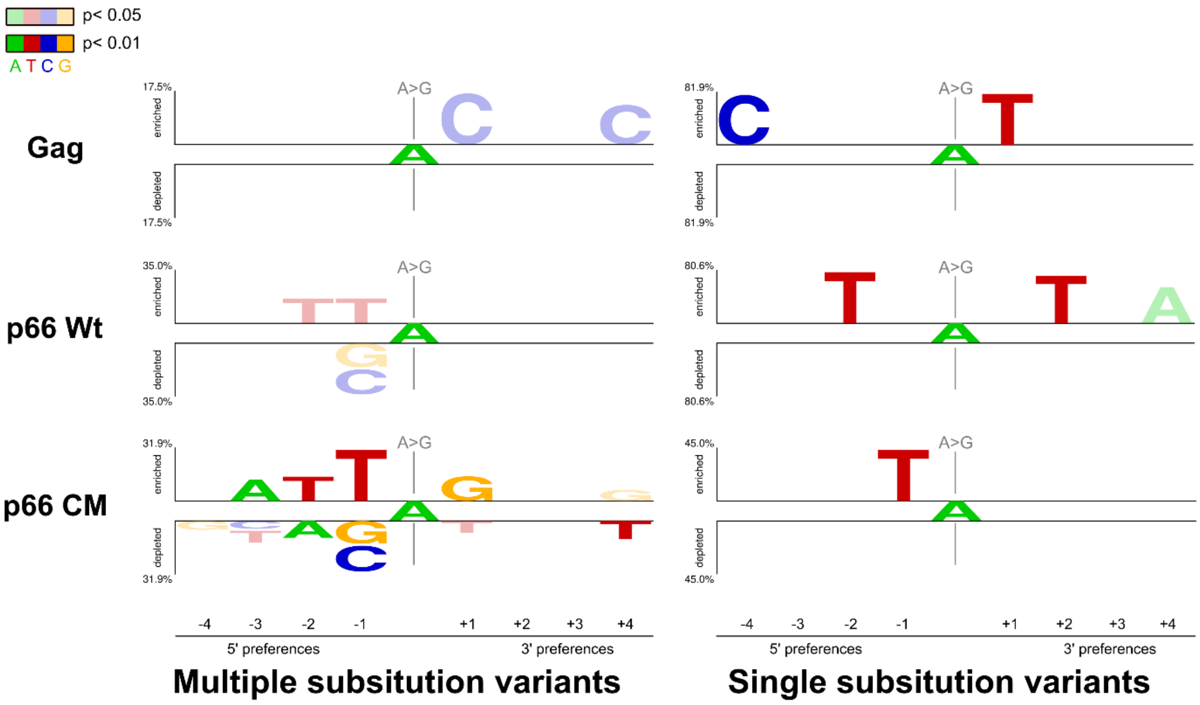

Figure S1. Two Sample logos of HIV-1 Gag, protease, RT p66 (p66 Wt) and p66 Codon Mutated RT (p66 CM) illustrating the underlying sequence contexts of all adenosine mutations. Bases were coloured opaque when $p < 0.01$, and translucent when $p < 0.05$.

```
ATGGATGGACCAAAAGTAAAACAATGGCCTTTAACAGAAGAAAAAATTAAAGCATTAGTAGAAATTTGTACAGAAATGGAAAAAGAAGGAAAAATTTCAA
AAATTGGTCCAGAAAATCCTTATAATACACCAGTATTTGCTATTAAGAAAAAAGATAGTACAAAATGGCGTAAATTAGTTGATTTTCGTGAATTAAATAA
AAGAACACAAGATTTTTGGGAAGTACAATTAGGTATTCCACATCCTGCAGGTTTAAAAAAGAAAAAATCTGTAACAGTTTTAGATGTAGGAGATGCATAT
TTTTCAGTTCCATTAGATGAAGATTTTCGTAAATATACAGCTTTTACAATTCCATCTATTAATAATGAAACACCTGGTATTAGATATCAATATAATGTAT
TACCACAAGGATGGAAAGGTTCACCTGCAATTTTTCAATCTTCAATGACAAAAATTTTAGAACCATTTCGTAAACAAAATCCTGATATTGTAATTTATCA
ATATATGGATGATTTATATGTTGGATCTGATTTAGAAATTGGTCAACATAGAACAAAAATTGAAGAATTACGTCAACATTTATTAAGATGGGGATTAACA
ACACCAGATAAAAAACATCAAAAAGAACCACCATTTTTATGGATGGGTTATGAATTACATCCTGATAAATGGACAGTACAACCAATTGTTTTACCTGAAA
AAGATAGTTGGACAGTAAATGATATTCAAAAATTAGTTGGAAAACTTAATTGGGCATCTCAAATTTATCCAGGTATTAAAGTTCGTCAATTATGTAAACT
TTTAAGAGGAACAAAAGCATTAACAGAAGTAATTCCATTAACAGAAGAAGCAGAATTAGAATTAGCTGAAAATCGTGAAATTTTAAAAGAACCAGTACAT
GGTGTTTATTATGATCCTAGTAAAGATTTAATTGCAGAAATTCAAAAACAAGGACAAGGTCAATGGACATATCAAATTTATCAAGAACCATTTAAAAATT
TAAAAACAGGAAAATATGCACGTATGAGAGGTGCTCATACAAATGATGTAAAACAATTAACAGAAGCAGTTCAAAAAATTACAACAGAATCTATTGTTAT
TTGGGGAAAAACACCAAAATTTAAACTTCCTATTCAAAAAGAAACATGGGAAACATGGTGGACAGAATATTGGCAAGCTACATGGATTCCAGAATGGGAA
TTTGTAAATACACCACCTTTAGTTAAACTTTGGTATCAATTAGAAAAAGAACCTATTGTAGGAGCAGAAACATTTTATGTTGATGGTGCAGCTAATAGAG
AAACAAAACTTGGAAAAGCTGGTTATGTAACAAATCGTGGTAGACAAAAAGTAGTTACATTAACAGATACAACAAATCAAAAAACAGAATTACAAGCAAT
TTATTTAGCTTTACAAGATTCAGGATTAGAAGTAAATATTGTTACAGATAGTCAATATGCATTAGGTATTATTCAAGCTCAACCAGATCAAAGTGAATCT
GAATTAGTTAATCAAATTATTGAACAATTAATTAAGAAAGAAAAAGTATATTTAGCATGGGTTCCAGCTCATAAAGGAATTGGAGGTAATGAACAAGTAG
ATAAACTTGTAAGTGCTGGTATTAGAAAAGTATTA
```

Data S1. Nucleotide sequence of codon mutated RT p66.

References

1. Sanchez-Merino, V.; Farrow, M.A.; Brewster, F.; Somasundaran, M.; Luzuriaga, K. Identification and Characterization of HIV-1 CD8+ T Cell Escape Variants with Impaired Fitness. *The Journal of Infectious Diseases* **2008**, *197*, 300-308, doi:10.1086/524845.
2. Sanchez-Merino, V.; Nie, S.; Luzuriaga, K. HIV-1-Specific CD8+ T Cell Responses and Viral Evolution in Women and Infants. *The Journal of Immunology* **2005**, *175*, 6976-6986, doi:10.4049/jimmunol.175.10.6976.
3. Amano, M.; Tojo, Y.; Salcedo-Gómez, P.M.; Campbell, J.R.; Das, D.; Aoki, M.; Xu, C.-X.; Rao, K.V.; Ghosh, A.K.; Mitsuya, H. GRL-0519, a Novel Oxatricyclic Ligand-Containing Nonpeptidic HIV-1 Protease Inhibitor (PI), Potently Suppresses Replication of a Wide Spectrum of Multi-PI-Resistant HIV-1 Variants In Vitro. *Antimicrobial Agents and Chemotherapy* **2013**, *57*, 2036-2046, doi:10.1128/aac.02189-12.
4. Ristic, N.; Chin, M.P.S. Mutations in matrix and SP1 repair the packaging specificity of a Human Immunodeficiency Virus Type 1 mutant by reducing the association of Gag with spliced viral RNA. *Retrovirology* **2010**, *7*, 73, doi:10.1186/1742-4690-7-73.
5. Rihn, S.J.; Wilson, S.J.; Loman, N.J.; Alim, M.; Bakker, S.E.; Bhella, D.; Gifford, R.J.; Rixon, F.J.; Bieniasz, P.D. Extreme Genetic Fragility of the HIV-1 Capsid. *PLOS Pathogens* **2013**, *9*, e1003461, doi:10.1371/journal.ppat.1003461.
6. Shi, J.; Zhou, J.; Halambage, U.D.; Shah, V.B.; Burse, M.J.; Wu, H.; Blair, W.S.; Butler, S.L.; Aiken, C. Compensatory Substitutions in the HIV-1 Capsid Reduce the Fitness Cost Associated with Resistance to a Capsid-Targeting Small-Molecule Inhibitor. *Journal of Virology* **2015**, *89*, 208-219, doi:10.1128/jvi.01411-14.
7. von Schwedler, U.K.; Stray, K.M.; Garrus, J.E.; Sundquist, W.I. Functional Surfaces of the Human Immunodeficiency Virus Type 1 Capsid Protein. *Journal of Virology* **2003**, *77*, 5439-5450, doi:10.1128/jvi.77.9.5439-5450.2003.
8. De Iaco, A.; Luban, J. Cyclophilin A promotes HIV-1 reverse transcription but its effect on transduction correlates best with its effect on nuclear entry of viral cDNA. *Retrovirology* **2014**, *11*, 11, doi:10.1186/1742-4690-11-11.
9. Brockman, M.A.; Schneidewind, A.; Lahaie, M.; Schmidt, A.; Miura, T.; DeSouza, I.; Ryvkin, F.; Derdeyn, C.A.; Allen, S.; Hunter, E., et al. Escape and Compensation from Early HLA-B57-Mediated Cytotoxic T-Lymphocyte Pressure on Human Immunodeficiency Virus Type 1 Gag Alter Capsid Interactions with Cyclophilin A. *Journal of Virology* **2007**, *81*, 12608-12618, doi:10.1128/JVI.01369-07.
10. Schneidewind, A.; Tang, Y.; Brockman, M.A.; Ryland, E.G.; Dunkley-Thompson, J.; Steel-Duncan, J.C.; St. John, M.A.; Conrad, J.A.; Kalams, S.A.; Noel, F., et al. Maternal Transmission of Human Immunodeficiency Virus Escape Mutations Subverts HLA-B57 Immunodominance but Facilitates Viral Control in the Haploidentical Infant. *Journal of Virology* **2009**, *83*, 8616-8627, doi:10.1128/JVI.00730-09.
11. Liu, D.; Zuo, T.; Hora, B.; Song, H.; Kong, W.; Yu, X.; Goonetilleke, N.; Bhattacharya, T.; Perelson, A.S.; Haynes, B.F., et al. Preexisting compensatory amino acids compromise fitness costs of a HIV-1 T cell escape mutation. *Retrovirology* **2014**, *11*, 101, doi:10.1186/s12977-014-0101-0.
12. Bailey , J.R.; Williams , T.M.; Siliciano , R.F.; Blankson , J.N. Maintenance of viral suppression in HIV-1–infected HLA-B*57+ elite suppressors despite CTL escape mutations. *Journal of Experimental Medicine* **2006**, *203*, 1357-1369, doi:10.1084/jem.20052319.
13. van Bockel, D.J.; Price, D.A.; Munier, M.L.; Venturi, V.; Asher, T.E.; Ladell, K.; Greenaway, H.Y.; Zaunders, J.; Douek, D.C.; Cooper, D.A., et al. Persistent Survival of Prevalent Clonotypes within an Immunodominant HIV Gag-Specific CD8+ T Cell Response. *The Journal of Immunology* **2011**, *186*, 359-371, doi:10.4049/jimmunol.1001807.
14. Dick, R.A.; Zadrozny, K.K.; Xu, C.; Schur, F.K.M.; Lyddon, T.D.; Ricana, C.L.; Wagner, J.M.; Perilla, J.R.; Ganser-Pornillos, B.K.; Johnson, M.C., et al. Inositol phosphates are assembly co-factors for HIV-1. *Nature* **2018**, *560*, 509-512, doi:10.1038/s41586-018-0396-4.
15. Banke, S.; Lillemark, M.R.; Gerstoft, J.; Obel, N.; Jørgensen, L.B. Positive Selection Pressure Introduces Secondary Mutations at Gag Cleavage Sites in Human Immunodeficiency Virus Type 1 Harboring Major Protease Resistance Mutations. *Journal of Virology* **2009**, *83*, 8916-8924, doi:10.1128/jvi.00003-09.
16. Nijhuis, M.; van Maarseveen, N.M.; Lastere, S.; Schipper, P.; Coakley, E.; Glass, B.; Rovenska, M.; de Jong, D.; Chappey, C.; Goedegebuure, I.W., et al. A Novel Substrate-Based HIV-1 Protease Inhibitor Drug Resistance Mechanism. *PLOS Medicine* **2007**, *4*, e36, doi:10.1371/journal.pmed.0040036.
17. Yates, P.J.; Hazen, R.; St. Clair, M.; Boone, L.; Tisdale, M.; Elston, R.C. In Vitro Development of Resistance to Human Immunodeficiency Virus Protease Inhibitor GW640385. *Antimicrobial Agents and Chemotherapy* **2006**, *50*, 1092-1095, doi:10.1128/aac.50.3.1092-1095.2006.

18. Nomura, S.; Hosoya, N.; Brumme, Z.L.; Brockman, M.A.; Kikuchi, T.; Koga, M.; Nakamura, H.; Koibuchi, T.; Fujii, T.; Carlson, J.M., et al. Significant Reductions in Gag-Protease-Mediated HIV-1 Replication Capacity during the Course of the Epidemic in Japan. *Journal of Virology* **2013**, *87*, 1465-1476, doi:10.1128/jvi.02122-12.
19. Blais, M.-E.; Zhang, Y.; Rostron, T.; Griffin, H.; Taylor, S.; Xu, K.; Yan, H.; Wu, H.; James, I.; John, M., et al. High frequency of HIV mutations associated with HLA-C suggests enhanced HLA-C-restricted CTL selective pressure associated with an AIDS-protective polymorphism. *J Immunol* **2012**, *188*, 4663-4670, doi:10.4049/jimmunol.1103472.
20. Kim, S.; Kim, Y.-C.; Qi, H.; Su, K.; Morrison, S.L.; Chow, S.A. Efficient Identification of Human Immunodeficiency Virus Type 1 Mutants Resistant to a Protease Inhibitor by Using a Random Mutant Library. *Antimicrobial Agents and Chemotherapy* **2011**, *55*, 5090-5098, doi:10.1128/aac.00687-11.
21. Mohamed, S.; Penaranda, G.; Gonzalez, D.; Camus, C.; Khiri, H.; Boulmé, R.; Sayada, C.; Philibert, P.; Olive, D.; Halfon, P. Comparison of ultra-deep versus Sanger sequencing detection of minority mutations on the HIV-1 drug resistance interpretations after virological failure. *AIDS* **2014**, *28*, 1315-1324, doi:10.1097/qad.0000000000000267.
22. Fisher, T.S.; Darden, T.; Prasad, V.R. Substitutions at Phe61 in the β3-β4 Hairpin of HIV-1 Reverse Transcriptase Reveal a Role for the Fingers Subdomain in Strand Displacement DNA Synthesis. *J Mol Biol* **2003**, *325*, 443-459, doi:10.1016/S0022-2836(02)01225-1.
23. Ceccherini-Silberstein, F.; Gago, F.; Santoro, M.; Gori, C.; Svicher, V.; Rodríguez-Barrios, F.; d'Arrigo, R.; Ciccozzi, M.; Bertoli, A.; Monforte, A.d.A., et al. High Sequence Conservation of Human Immunodeficiency Virus Type 1 Reverse Transcriptase under Drug Pressure despite the Continuous Appearance of Mutations. *Journal of Virology* **2005**, *79*, 10718-10729, doi:10.1128/jvi.79.16.10718-10729.2005.
24. Auwerx, J.; Van Nieuwenhove, J.; Rodríguez-Barrios, F.; de Castro, S.; Velázquez, S.; Ceccherini-Silberstein, F.; De Clercq, E.; Camarasa, M.-J.; Perno, C.-F.; Gago, F., et al. The N137 and P140 amino acids in the p51 and the P95 amino acid in the p66 subunit of human immunodeficiency virus type 1 (HIV-1) reverse transcriptase are instrumental to maintain catalytic activity and to design new classes of anti-HIV-1 drugs. *FEBS Letters* **2005**, *579*, 2294-2300, doi:10.1016/j.febslet.2005.02.077.
25. Kamangu, E.N. Correlation between Mutations at the Beginning of Treatment and Virological Failure after 6 Months of Antiretroviral Treatment in People Living with the Human Immunodeficiency Virus in Kinshasa. *ARC Journal of AIDS* **2018**, *3*, 22-27.
26. Mackie, N. Resistance to non-nucleoside reverse transcriptase inhibitors. In *Antiretroviral resistance in clinical practice*, Mediscript: 2006.
27. Derache, A.; Maiga, A.-I.; Traore, O.; Akonde, A.; Cisse, M.; Jarrousse, B.; Koita, V.; Diarra, B.; Carcelain, G.; Barin, F., et al. Evolution of genetic diversity and drug resistance mutations in HIV-1 among untreated patients from Mali between 2005 and 2006. *Journal of Antimicrobial Chemotherapy* **2008**, *62*, 456-463, doi:10.1093/jac/dkn234.
28. Johnson, V.A.; Brun-Vezinet, F.; Clotet, B.; Gunthard, H.F.; Kuritzkes, D.R.; Pillay, D.; Schapiro, J.M.; Richman, D.D. Update of the Drug Resistance Mutations in HIV-1. *Top HIV Med* **2008**, *16*, 138-145.
29. Wu, H.; Zhang, H.-J.; Zhang, X.-m.; Xu, H.-f.; Wang, M.; Huang, J.-d.; Zheng, B.-J. Identification of Drug Resistant Mutations in HIV-1 CRF07_BC Variants Selected by Nevirapine In Vitro. *PLOS ONE* **2012**, *7*, e44333, doi:10.1371/journal.pone.0044333.
30. Parkin, N.T.; Gupta, S.; Chappey, C.; Petropoulos, C.J. The K101P and K103R/V179D Mutations in Human Immunodeficiency Virus Type 1 Reverse Transcriptase Confer Resistance to Nonnucleoside Reverse Transcriptase Inhibitors. *Antimicrobial Agents and Chemotherapy* **2006**, *50*, 351-354, doi:10.1128/aac.50.1.351-354.2006.
31. Neogi, U.; Shet, A.; Shamsundar, R.; Ekstrand, M.L. Selection of nonnucleoside reverse transcriptase inhibitor-associated mutations in HIV-1 subtype C: evidence of etravirine cross-resistance. *AIDS (London, England)* **2011**, *25*, 1123-1126, doi:10.1097/QAD.0b013e328346269f.
32. Ren, J.; Nichols, C.E.; Chamberlain, P.P.; Weaver, K.L.; Short, S.A.; Stammers, D.K. Crystal Structures of HIV-1 Reverse Transcriptases Mutated at Codons 100, 106 and 108 and Mechanisms of Resistance to Non-nucleoside Inhibitors. *J Mol Biol* **2004**, *336*, 569-578, doi:10.1016/j.jmb.2003.12.055.
33. Balzarini, J.; Auwerx, J.; Rodríguez-Barrios, F.; Chedad, A.; Farkas, V.; Ceccherini-Silberstein, F.; García-Aparicio, C.; Velázquez, S.; De Clercq, E.; Perno, C.-F., et al. The Amino Acid Asn136 in HIV-1 Reverse Transcriptase (RT) Maintains Efficient Association of Both RT Subunits and Enables the Rational Design of Novel RT Inhibitors. *Molecular Pharmacology* **2005**, *68*, 49-60, doi:10.1124/mol.105.012435.
34. Thorat, S.R.; Chaturbhuj, D.N.; Hingankar, N.K.; Chandrasekhar, V.; Koppada, R.; Datkar, S.R.; Srikantiah, P.; Garg, R.; Kabra, S.; Haldar, P., et al. Surveillance of Transmitted HIV Type 1 Drug Resistance Among

HIV Type 1-Positive Women Attending an Antenatal Clinic in Kakinada, India. *AIDS Research and Human Retroviruses* **2011**, *27*, 1291-1297, doi:10.1089/aid.2011.0036.
35. Hayashida, T.; Hachiya, A.; Ode, H.; Nishijima, T.; Tsuchiya, K.; Sugiura, W.; Takiguchi, M.; Oka, S.; Gatanaga, H. Rilpivirine resistance mutation E138K in HIV-1 reverse transcriptase predisposed by prevalent polymorphic mutations. *Journal of Antimicrobial Chemotherapy* **2016**, *71*, 2760-2766, doi:10.1093/jac/dkw224.
36. Zhang, X.-M.; Zhang, Q.; Wu, H.; Lau, T.C.-K.; Liu, X.; Chu, H.; Zhang, K.; Zhou, J.; Chen, Z.-W.; Jin, D.-Y., et al. Novel Mutations L228I and Y232H Cause Nonnucleoside Reverse Transcriptase Inhibitor Resistance in Combinational Pattern. *AIDS Research and Human Retroviruses* **2016**, *32*, 909-917, doi:10.1089/aid.2015.0359.
37. Shahriar, R.; Rhee, S.-Y.; Liu, T.F.; Fessel, W.J.; Scarsella, A.; Towner, W.; Holmes, S.P.; Zolopa, A.R.; Shafer, R.W. Nonpolymorphic Human Immunodeficiency Virus Type 1 Protease and Reverse Transcriptase Treatment-Selected Mutations. *Antimicrobial Agents and Chemotherapy* **2009**, *53*, 4869-4878, doi:10.1128/aac.00592-09.
38. Saravanan, S.; Madhavan, V.; Solomon, S.S.; Kantor, R.; Katzenstein, D.; Sivamalar, S.; Kumarasamy, N.; Smith, D.M.; Mayer, K.H.; Solomon, S., et al. Reverse Transcriptase Substitution at Codons 208 and 228 Among Treatment-Experienced HIV-1 Subtype-C–Infected Indian Patients Is Strongly Associated With Thymidine Analogue Mutations. *JAIDS Journal of Acquired Immune Deficiency Syndromes* **2012**, *59*, e26-e27, doi:10.1097/QAI.0b013e31823e2d2b.
39. Rath, B.A.; Yousef, K.P.; Katzenstein, D.K.; Shafer, R.W.; Schütte, C.; von Kleist, M.; Merigan, T.C. In Vitro HIV-1 Evolution in Response to Triple Reverse Transcriptase Inhibitors & In Silico Phenotypic Analysis. *PLOS ONE* **2013**, *8*, e61102, doi:10.1371/journal.pone.0061102.
40. Marcelin, A.-G.; Flandre, P.; Furco, A.; Wirden, M.; Molina, J.-M.; Calvez, V.; Team, A.I.J.S. Impact of HIV-1 reverse transcriptase polymorphism at codons 211 and 228 on virological response to didanosine. *Antivir Ther* **2006**, *11*, 693-699.
41. Gonzales, M.J.; Wu, T.D.; Taylor, J.; Belitskaya, I.; Kantor, R.; Israelski, D.; Chou, S.; Zolopa, A.R.; Fessel, W.J.; Shafer, R.W. Extended spectrum of HIV-1 reverse transcriptase mutations in patients receiving multiple nucleoside analog inhibitors. *AIDS* **2003**, *17*, 791-799.
42. Ceccherini-Silberstein, F.; Svicher, V.; Sing, T.; Artese, A.; Santoro, M.M.; Forbici, F.; Bertoli, A.; Alcaro, S.; Palamara, G.; d'Arminio Monforte , A., et al. Characterization and Structural Analysis of Novel Mutations in Human Immunodeficiency Virus Type 1 Reverse Transcriptase Involved in the Regulation of Resistance to Nonnucleoside Inhibitors. *Journal of Virology* **2007**, *81*, 11507-11519, doi:10.1128/jvi.00303-07.
43. Hachiya, A.; Kodama, E.N.; Sarafianos, S.G.; Schuckmann, M.M.; Sakagami, Y.; Matsuoka, M.; Takiguchi, M.; Gatanaga, H.; Oka, S. Amino Acid Mutation N348I in the Connection Subdomain of Human Immunodeficiency Virus Type 1 Reverse Transcriptase Confers Multiclass Resistance to Nucleoside and Nonnucleoside Reverse Transcriptase Inhibitors. *Journal of Virology* **2008**, *82*, 3261-3270, doi:10.1128/jvi.01154-07.
44. Santos, A.F.A.; Lengruber, R.B.; Soares, E.A.; Jere, A.; Sprinz, E.; Martinez, A.M.B.; Silveira, J.; Sion, F.S.; Pathak, V.K.; Soares, M.A. Conservation Patterns of HIV-1 RT Connection and RNase H Domains: Identification of New Mutations in NRTI-Treated Patients. *PLOS ONE* **2008**, *3*, e1781, doi:10.1371/journal.pone.0001781.
45. Tachedjian, G.; Aronson, H.-E.G.; Santos, M.d.l.; Seehra, J.; McCoy, J.M.; Goff, S.P. Role of Residues in the Tryptophan Repeat Motif for HIV-1 Reverse Transcriptase Dimerization. *J Mol Biol* **2003**, *326*, 381-396, doi:10.1016/S0022-2836(02)01433-X.
46. Kemp, S.D.; Shi, C.; Bloor, S.; Harrigan, P.R.; Mellors, J.W.; Larder, B.A. A Novel Polymorphism at Codon 333 of Human Immunodeficiency Virus Type 1 Reverse Transcriptase Can Facilitate Dual Resistance to Zidovudine and L-2′,3′-Dideoxy-3′-Thiacytidine. *Journal of Virology* **1998**, *72*, 5093-5098.
47. Grohmann, D.; Corradi, V.; Elbasyouny, M.; Baude, A.; Horenkamp, F.; Laufer, S.D.; Manetti, F.; Botta, M.; Restle, T. Small Molecule Inhibitors Targeting HIV-1 Reverse Transcriptase Dimerization. *ChemBioChem* **2008**, *9*, 916-922, doi:10.1002/cbic.200700669.
48. Roquebert, B.; Wirden, M.; Simon, A.; Deval, J.; Katlama, C.; Calvez, V.; Marcelin, A.-G. Relationship between mutations in HIV-1 RNase H domain and nucleoside reverse transcriptase inhibitors resistance mutations in naïve and pre-treated HIV infected patients. *Journal of Medical Virology* **2007**, *79*, 207-211, doi:10.1002/jmv.20788.
49. Bailey, J.R.; O'Connell, K.; Yang, H.-C.; Han, Y.; Xu, J.; Jilek, B.; Williams, T.M.; Ray, S.C.; Siliciano, R.F.; Blankson, J.N. Transmission of Human Immunodeficiency Virus Type 1 from a Patient Who Developed AIDS to an Elite Suppressor. *Journal of Virology* **2008**, *82*, 7395-7410, doi:10.1128/jvi.00800-08.
